# Reliability and validity of rapid assessment tools for measuring 24-hour movement behaviours in children aged 0–5 years: the Movement Behaviour Questionnaire Baby (MBQ-B) and child (MBQ-C)

**DOI:** 10.1186/s12966-024-01596-5

**Published:** 2024-04-23

**Authors:** Stewart G. Trost, Caroline O. Terranova, Denise S.K. Brookes, Li Kheng Chai, Rebecca A. Byrne

**Affiliations:** 1 School of Human Movement and Nutrition Sciences, The University of Queensland, Brisbane, Australia; 2 School of Exercise and Nutrition Sciences, Queensland University of Technology, Brisbane, Australia; 3 Health and Wellbeing Queensland, Queensland Government, Brisbane, Australia

**Keywords:** Physical activity, Active play, Screen time, Sleep, Infants, Toddlers, Preschoolers, Self-report, Survey, Assessment

## Abstract

**Background:**

The development of validated “fit-for-purpose” rapid assessment tools to measure 24-hour movement behaviours in children aged 0–5 years is a research priority. This study evaluated the test-retest reliability and concurrent validity of the open-ended and closed-ended versions of the Movement Behaviour Questionnaire for baby (MBQ-B) and child (MBQ-C).

**Methods:**

300 parent-child dyads completed the 10-day study protocol (MBQ-B: *N* = 85; MBQ-C: *N* = 215). To assess validity, children wore an accelerometer on the non-dominant wrist (ActiGraph GT3X+) for 7 days and parents completed 2 × 24-hour time use diaries (TUDs) recording screen time and sleep on two separate days. For babies (i.e., not yet walking), parents completed 2 × 24-hour TUDs recording tummy time, active play, restrained time, screen time, and sleep on days 2 and 5 of the 7-day monitoring period. To assess test-retest reliability, parents were randomised to complete either the open- or closed-ended versions of the MBQ on day 7 and on day 10. Test-retest intraclass correlation coefficients (ICC’s) were calculated using generalized linear mixed models and validity was assessed via Spearman correlations.

**Results:**

Test-retest reliability for the MBQ-B was good to excellent with ICC’s ranging from 0.80 to 0.94 and 0.71–0.93 for the open- and closed-ended versions, respectively. For both versions, significant positive correlations were observed between 24-hour diary and MBQ-B reported tummy time, active play, restrained time, screen time, and sleep (rho = 0.39–0.87). Test-retest reliability for the MBQ-C was moderate to excellent with ICC’s ranging from 0.68 to 0.98 and 0.44–0.97 for the open- and closed-ended versions, respectively. For both the open- and closed-ended versions, significant positive correlations were observed between 24-hour diary and MBQ-C reported screen time and sleep (rho = 0.44–0.86); and between MBQ-C reported and device-measured time in total activity and energetic play (rho = 0.27–0.42).

**Conclusions:**

The MBQ-B and MBQ-C are valid and reliable rapid assessment tools for assessing 24-hour movement behaviours in infants, toddlers, and pre-schoolers. Both the open- and closed-ended versions of the MBQ are suitable for research conducted for policy and practice purposes, including the evaluation of scaled-up early obesity prevention programs.

**Supplementary Information:**

The online version contains supplementary material available at 10.1186/s12966-024-01596-5.

## Introduction

It is widely acknowledged that the right combination of physical activity, sedentary time, and sleep is essential for healthy growth and development in early life [1]. Regular participation in physical activity, limited sedentary screen time, and sufficient levels of quality sleep are associated with a myriad of important outcomes, including cardiometabolic health, bone health, motor development, as well as multiple indicators of social, cognitive, and emotional development [2–7]. Based on this evidence, the World Health Organization issued 24-hour movement guidelines delineating the minimum amount of time in a 24-hour day that young children under 5 years of age should spend being physically active, optimal duration of sleep, and the maximum recommended time children should spend in screen-based sedentary activities or time restrained (e.g., stroller or car seat) [8]. Notably, 24-hour movement guidelines for children aged 0 to 5 years have also been issued by public health organisations in Australia [9], Canada [10], South Africa [11], and the United Kingdom [12].

With the release of such guidelines, there is an urgent need for validated “fit-for-purpose” assessment tools to measure 24-hour movement behaviours in children aged 0–5 years [8, 9, 13]. Valid and reliable measures of young children’s movement behaviours are necessary to monitor population-level trends in movement behaviours, evaluate scaled-up interventions to promote healthy movement behaviours, and make informed public health policy and practice decisions [14]. While device-based assessments of movement behaviours (i.e., accelerometers) are preferred and frequently employed in studies of young children, such methods are difficult to implement in policy and practice settings as well as large population-based studies [15, 16]. In addition, while wearable sensors can measure sedentary behaviour, they do not directly measure children’s screen time exposures. Currently, valid and/or reliable proxy-report questionnaires for assessing physical activity, sedentary behaviour, and sleep in young children aged 0 to 5 years are lacking. Our systematic review of brief tools for measuring obesity-related behaviours in young children [17] concluded that currently available proxy-report tools for young children have limited validity and reliability and do not consider the large variations in movement behaviours across developmental periods. More importantly, there were no brief tools available for use with young children that could both validly and reliably measure all three movement behaviours included in the 24-hour movement guidelines - physical activity, sedentary behaviour (including screen time), and sleep. Similar conclusions were reached in two recently published systematic reviews of questionnaires measuring 24-hour movement behaviours in young children [18, 19]. To address this gap in the research literature, the objective of the current study was to evaluate the test-retest reliability and concurrent validity of two newly developed short-form measurement tools designed to rapidly assess 24-hour movement behaviours in children aged 0–5 years: the Movement Behaviour Questionnaire - Baby (MBQ-B) and Movement Behaviour Questionnaire - Child (MBQ-C).

## Methods

### Development of Movement Behaviour questionnaires

Based on systematic reviews of existing measures [17, 20] and an extensive cognitive interviewing study with parents and caregivers [21], separate questionnaires were developed for babies and children. The MBQ-B comprises six items in total, with one item each for tummy time or active floor-based play, restrained time, non-interactive screen time, interactive screen time, night-time sleep, and daytime sleep. The MBQ-C comprises nine items in total, two for active play, two for non-interactive screen time, two for interactive screen time, and three for sleep. The MBQ-C included two items each for active play, non-interactive screen time, and interactive screen time because of separate items for weekdays and weekends. The three items for sleep addressed night-time sleep, daytime sleep, and sleep regularity. The active play items included a sub-item for determination of the amount of time engaged in energetic play or vigorous intensity activities, while the non-interactive and interactive screen time items included a sub-item related to the child’s posture during sedentary screen time. Copies of the MBQ instruments and detailed instructions for calculating summary variables are provided in supplementary material 2 and can be found on https://research.qut.edu.au/cparg/projects/movement-behaviour-questionnaire-mbq-screening-tools/.

The types of physical activities performed by children aged 0 to 5 years are dependent on motor development milestones (i.e., the child’s ability to roll over, sit without support, crawl, stand without support, and walk). Thus, the decision on which MBQ tool to use is based on developmental milestones rather than the child’s age. Parents or caregivers with children who have reached their walking milestone complete the MBQ-C. Within the MBQ-C, the active play items ask parents to recall their child’s time spent in activities such as walking, running, dancing, and climbing. If children have not reached their walking milestone, parents or caregivers complete the MBQ-B. Within the MBQ-B, the active play item is also based on developmental milestones. If children have not reached their rolling milestone, parents/caregivers complete the items about tummy time. If their child has reached their rolling milestone, parents/caregivers complete the item about supervised floor-based play (e.g., crawling on the floor with your baby).

Both open-ended and closed-ended versions of the MBQ-B and MBQ-C were developed and evaluated. The open-ended version was created primarily for research applications where data is collected for statistical purposes and examined for differential change within or between groups over time. The closed-ended versions were considered a pragmatic option for use in clinical or primary care settings where the scores could provide immediate feedback and used for goal setting and self-monitoring purposes.

### Participants, recruitment, and randomisation

Parents with children aged 0–5 years residing in Australia were eligible to participate in the study. Parent-child dyads were recruited through a range of study promotion avenues. This included newsletters and/or flyers at childcare centres, family-friendly community venues (e.g., libraries, gyms, cafes), social media networks (e.g., targeted Facebook advertisements and parenting support groups), and word-of-mouth. Parents interested in the study were directed to an electronic registration link or QR Code (on printed materials) enabling them to access a copy of the Parent Information Sheet, contact the research team if they had any questions, and provide consent to participate. Consenting participants provided their contact details and completed a brief demographics survey recording parental age range, gender, indigenous status, language spoken at home, education level, postcode, child date of birth, height, weight, and the number of days their child attended childcare each week.

In total, 450 interested parents viewed the online Parent Information and Consent Form. Of these, 436 consented electronically to participate in the study. However, 127 of these parents were not contactable to begin data collection procedures (unable to be contacted or provided incorrect contact/incomplete details) and nine withdrew their consent, leaving a final sample of 300 parent-child dyads available for randomisation.

Parent-child dyads were randomised into one of four possible groups using a 2:2:1:1 allocation ratio. Groups differed on which version of the MBQ parents completed on Day 7 and Day 10 of the study protocol (see below). Group 1 completed the open-ended version on Day 7 and Day 10; Group 2 completed the closed-ended version on Day 7 and Day 10; Group 3 completed the open-ended version on Day 7 and the closed-ended version on Day 10; Group 4 completed the closed-ended version on Day 7 and the open-ended version on Day 10. Of the 85 parent-child dyads eligible to complete the MBQ-B, 28 were randomised to Group 1, 28 randomised to Group 2, 15 randomised to Group 3, and 14 randomised to Group 4. Of the 215 parent-child dyads eligible MBQ-C, 72 were randomised to Group 1, 72 randomised to Group 2, 35 randomised to Group 3, and 36 randomised to Group 4.

### Study protocol

Participants received a study pack via express mail containing the following: an introductory letter to parents, a detailed instruction sheet, and two 24-hour time-use diaries (TUDs). Parents/caregivers with children who had reached their walking milestone also received an accelerometer and an activity monitoring log sheet to record activity monitor removals. Study packs contained a reply-paid express mail envelope for the return of the accelerometer, monitoring log, and activity diaries.

Data collection took place over a 10-day period. Day one was defined as the first full day after receipt of the study pack. On Days 1 through 7, children who had reached their walking milestone wore an accelerometer on the non-dominant wrist 24 h a day. Children who had not yet reached their walking milestone did not wear an accelerometer. On Days 2 and 5, parents received text messages prompting them to complete a 24-hour TUD capturing detailed information about their child’s sleep, tummy time, active play, and screen time behaviours. The 24-hour TUDs were completed on Days 2 and 5 of the 10-day protocol to minimise participant burden and to ensure that the two 24-hour periods were non-contiguous and within the MBQ’s recall period (typical day over the past week). On Day 7, parents were prompted via text message to return the accelerometer and activity monitoring log sheet (children only) and the completed 24-hour time use diaries to the research team via the reply-paid express envelope. On Days 7 and 10, parents were sent a text message with a link to either the open- or closed-ended versions of the MBQ-B or MBQ-C to complete online. The first MBQ was completed on Day 7 to ensure that the recall period (a typical day in past week) overlapped with the 24-hour TUD and accelerometer data. The second MBQ was completed three days later on Day 10 of the protocol to evaluate test-retest reliability and agreement between different response formats (open vs. closed) for movement behaviours completed over the same recall period.

### Criterion measures

#### 24-hour time-use diary

Parents completed a 24-hour TUD designed to capture detailed information about their infant/child’s sleep, tummy time, active play, and screen time behaviours over a complete 24-hour cycle. Adapted from the 7-day screen time diary created by Mendoza and colleagues [22], the 24-hour period was segmented into 15-minute time blocks, organised into three 8-hour time periods corresponding to the “morning” (5:00 AM to 12:45 PM), “afternoon/evening” (1:00 PM to 8:45 PM), and “night” (9:00 PM to 4:45 AM). Parents/caregivers completing the MBQ-B were instructed to mark each 15-minute time block during which their infant’s main activity was sleeping, tummy time, supervised active play, restrained time, non-interactive screen time, or interactive screen time. Parents/caregivers completing the MBQ-C were instructed to mark each 15-minute time block during which their child’s main activity was sleeping, non-interactive screen time, or interactive screen time. Parents/caregivers completing the MBQ-C were also asked to identify the 15-minute blocks of screen time in which their child was standing. Descriptions of each movement behaviour were identical to those used in the MBQ-B and MBQ-C questionnaires. Time spent in each activity was calculated by counting the number 15-minute blocks marked by the parent and multiplying by 15. Estimates for Day 2 and Day 5 were averaged. The 24-hour TUDs are included in supplementary material 3.

#### Device-measured physical activity

For children who had reached their walking milestone, physical activity was measured using the ActiGraph GT3X + accelerometer (ActiGraph Corporation, Pensacola FL, USA). The physical activity metrics were the average magnitude of wrist acceleration (indicative of the daily volume of physical activity), average daily time spent in physical activity of any intensity (total physical activity), and average daily time spent in moderate-to-vigorous physical activity (MVPA).

The average magnitude of dynamic wrist acceleration was calculated using the Euclidian norm minus one gravitational unit (ENMO) [23]. ENMO units were derived by calculating the vector magnitude of the raw acceleration signal in each axis and subtracting 1 (to correct for the static component of gravity). Negative values were rounded up to zero. Prior to calculating ENMO, the raw acceleration signal was calibrated to local gravity using the in-situ autocalibration procedures described by Nadeau et al. [24].

Total daily physical activity and time spent in MVPA estimates were generated using a random forest physical activity classification model specifically trained on children age five years and younger [25]. This validated machine learning model uses over 20 features extracted from the raw tri-axial acceleration signal to classify activity type and quantify daily time spent in sedentary activities (sitting or lying down), light-intensity activities and games (slow walking, standing, standing arts and crafts), walking, running, and moderate-to-vigorous intensity activities and games (active games with balls, riding bikes/scooters). In a free-living evaluation, the random forest classification model exhibited significantly higher agreement with measured physical activity intensity than cut-points methods and exhibited evidence of equivalence with directly observed time in sedentary activity, light-intensity physical activity, and MVPA [26]. Total physical activity was calculated by summing daily time spent in light-intensity activities and games, walking, running, and moderate-to-vigorous activities and games; while MVPA was calculated by summing daily time spent in walking, running, and moderate-to-vigorous activities and games. Non-wear periods were identified by summing the 15 s windows in which the standard deviation of the vector magnitude was < 13 mg for > = 30 consecutive minutes [27]. Accelerometer data was included in the analyses if they had ≥ 4 days in which wear time was 960 min or longer.

### Data reduction

Responses to the open-ended versions of the MBQ were recorded in hours and minutes and converted to minutes per day. Responses to the closed-ended versions of the MBQ were expressed in minutes per day based on the mid-point of the selected response category. For the psychometric evaluation, extreme or implausible values for tummy time (> 180 min/day), total active play (> 480 min/day), energetic play (> 360 min/day), total restrained time (> 360 min/day), passive or interactive screen time (> 600 min/day), and daytime sleep (> 360 min/day, MBQ-C only) were considered non-valid responses. For the MBQ-C, responses to the weekday and weekend items were combined to a daily metric by calculating the weighted average as follows: ([Weekday response x 5] + [Weekend day response x 2] / 7).

### Statistical analysis

Descriptive characteristics for the sample were presented as means and standard deviations for continuous variables and as percentages for categorical variables. For parents completing the same version of the MBQ on Day 7 and Day 10 (Groups 1 and 2), test-retest reliability was evaluated using intraclass correlation coefficients (ICCs). For parents completing a different version of the MBQ on Day 7 and Day 10 (Groups 3 and 4), agreement between open- and closed-ended responses were evaluated using spearman rank order correlation coefficients and ICCs. Associations between MBQ measured outcomes and the corresponding criterion measures (24-hour TUD time and device-based physical activity) were assessed using Spearman rank order correlations coefficients. These correlations were calculated using the responses to the version of the MBQ completed on Day 7 of the 10-day protocol. Thus, validity coefficients for the open-ended versions of the MBQ were calculated from the Day 7 responses provided by parents randomised to Groups 1 and 3, while validity coefficients for the closed-ended versions of the MBQ were calculated from the Day 7 responses provided by parents randomised to Groups 2 and 4.

Test-retest intraclass correlations (ICCs) were calculated using generalised linear mixed-effects models implemented via the rptR package in R [28]. Confidence intervals were estimated via parametric bootstrapping (1000 iterations). An ICC of 0.90 or higher was considered to represent an excellent level of agreement. ICCs of 0.75–0.89 were considered to represent a good level of agreement. ICC values of 0.50–0.74 were considered to represent moderate reliability, and those below 0.5 represented poor reliability [29]. Spearman correlations were calculated using the CORR procedure in SAS Version 9.2 (SAS Institute, Cary, NC). The resultant associations were interpreted using the ratings suggested by Schober et al. [30]: 0.00–0.10 (negligible correlation), 0.10–0.39 (weak correlation), 0.40–0.69 (moderate correlation), 0.70–0.89 (strong correlation), and 0.90–1.00 (very strong correlation) [30]. For all tests, statistical significance was set at alpha level of 0.05.

## Results

Table 1 reports the descriptive characteristics of the total sample and the parent-child dyads invited to complete the open- or closed-ended versions of MBQ-B and MBQ-C questionnaires, respectively. The majority of parent/caregiver respondents were female and between 26 and 35 years of age. The educational attainment profile was broadly representative of Australian women between 18 and 45 years, with approximately two-thirds of the sample completing a university degree [31]. Participating families resided in postcodes representing all 10 deciles of the Index of Relative Socio-economic Advantage and Disadvantage (IRSAD) [32], with approximately three quarters of the sample in deciles 5 through 10. A higher decile indicates a relative lack of disadvantage and greater advantage in general. The MBQ-B sample had approximately equal numbers of boys and girls, while the child sample had a higher proportion of male children. The mean age of the MBQ-B sample was 6.5 months, while the mean age of the MBQ-C sample was 38 months, or just over 3 years of age. Most children either did not attend childcare or attended 1 to 3 days per week. Based on parent reported height and weight, approximately 55% of children were in the healthy weight range, with approximately 17% of children affected by obesity or overweight.

**Table 1:** Descriptive characteristics of parents and children participating and for those in the MBQ-B and MBQ-C samples

	Total Sample (*N* = 300)	MBQ-B Sample (*N* = 85)	MBQ-C Sample (*N* = 215)
**Parent Respondent Age** (years)			
18–25	3.0%	4.7%	2.3%
26–35	56.0%	68.2%	51.2%
36–45	39.7%	27.1%	44.7%
> 45	1.3%	0.0%	1.9%
**Parent Respondent Education**			
High School Certificate	8.3%	11.8%	7.0%
TAFE/Diploma/Certificate	20.0%	21.2%	19.5%
University Undergraduate	35.7%	35.3%	35.8%
University Postgraduate	36.0%	31.8%	37.7%
**SEIFA Decile (IRSAD)**			
1–2 (most disadvantaged)	8.6%	8.2%	8.9%
3–4	14.6%	20.0%	12.4%
5–6	24.0%	21.0%	22.8%
7–8	27.5%	22.4%	29.3%
9–10 (least disadvantaged)	25.3%	22.4%	26.6%
**Child Sex**			
Female	46.7%	49.4%	45.6%
Male	53.3%	50.6%	54.4%
**Child Age (months)**	29.0 ± 18.9	6.5 ± 3.4	37.8 ± 14.8
**Childcare Attendance**			
0 days	25%	45.9%	16.7%
1–3 days/week	42%	36.5%	44.2%
4–5 days/week	33%	17.6%	39.1%
**Number of children in household aged 5 years or younger**			
1 Child	55.0%	62.4%	52.1%
2 Children	40.7%	34.1%	43.3%
≥ 3 Children	4.3%	3.5%	4.6%
**Child BMI z-score**	0.54 ± 1.7	0.30 ± 1.6	0.64 ± 1.7
Child Weight Status *			
Underweight	6.8%	9.3%	5.8%
Healthy weight	55.3%	58.7%	54.0%
At-risk of overweight	20.5%	18.7%	21.2%
Overweight	10.6%	8.0%	11.6%
Obese	6.8%	5.3%	7.4%

IRSAD = Index of Relative Socio-economic Advantage and Disadvantage

* WHO Child Growth Standards for Children 0–5 Years based on parent-reported height and weight

### Results for the MBQ-B

Of the 85 parent-child dyads, 77 (91%) completed at least one MBQ-B questionnaire, with 74 (87%) parents completing the two MBQ-B questionnaires as instructed on Day 7 and Day 10, respectively. Of the 77 parents completing one MBQ-B, 72 (85%) completed the 24-hour TUD. Within the sample, 14 parents had infants who had not reached their rolling milestone and completed the tummy time items. Of these 14 parents, 13 completed the tummy time items on Day 7 and Day 10, and 12 completed the 24-hour TUD. Compared to those completing all planned assessments, parents with missing data on at least one assessment tended to be older, less likely to have a university degree, less likely to have multiple children under the age of five and have a child attending childcare 4 to 5 days per week. However, there were no meaningful differences for relative socio-economic advantage and disadvantage, child sex, child age, and child weight status. (see supplementary material 1).

Table 2 reports the test-retest reliability results for the open- and closed-ended versions of the MBQ-B. ICCs for the open-ended version of the MBQ-B were good to excellent with ICCs ranging from 0.80 to 0.94. Reliability coefficients for the closed-ended version of the MBQ-B were good to excellent with ICC’s ranging from 0.71 to 0.93. Compared to the open-ended version, ICCs were marginally lower for tummy time, interactive screen time, and daytime sleep. Test-retest reliability for the closed-ended version of the tummy time item was not evaluated because only two parent-child dyads in Group 2 had not reach their rolling milestone and completed this item.

**Table 2:** Test-retest reliability coefficients and median duration for movement behaviours reported on Day 7 and 10 using the open- or closed-ended versions of the MBQ-B

MBQ-B Open				
		Reliability	Median (25th– 75th percentile)	
	N*	**ICC (95% CI)**	**Day 7 (mins/day)**	**Day 10 (mins/day)**
**Physical Activity**				
Tummy Time	6	0.92 (0.70–0.98)	18 (10–40)	30 (15–40)
Active Play	20	0.90 (0.76–0.96)	315 (240–450)	315 (270–420)
Restrained Time	26	0.90 (0.76–0.96)	130 (90–210)	120 (90–180)
**Screen Time**				
Non-interactive	27	0.80 (0.62–0.95)	10 (0–60)	10 (0–60)
Interactive	27	0.91 (0.75–0.99)	5 (0–60)	5 (0–60)
Total Screen	27	0.90 (0.80–0.97)	20 (0–90)	20 (5–60)
**Sleep**				
Day Sleep	27	0.94 (0.86–0.97)	150 (120–210)	180 (120–210)
Night Sleep	27	0.88 (0.74–0.94)	675 (600–720)	660 (600–690)
Total Sleep	27	0.87 (0.74–0.93)	840 (746–880)	830 (750–870)
**MBQ-B Closed**				
**Physical Activity**				
Tummy Time	2	--	--	--
Active Play	24	0.84 (0.68–0.93)	105 (75–120)	105 (75–120)
Restrained Time	24	0.80 (0.78–0.95)	105 (75–105)	90 (45–105)
**Screen Time**				
Non-interactive	26	0.93 (0.86–0.97)	0 (0–7.5)	0 (0–7.5)
Interactive	26	0.71 (0.49–0.92)	0 (0–7.5)	0 (0–7.5)
Total Screen	26	0.91 (0.81–0.96)	7.5 (0–15)	7.5 (0–15)
**Sleep**				
Day Sleep	26	0.73 (0.48–0.86)	150 (150–150)	150 (150–210)
Night Sleep	26	0.89 (0.76–0.93)	660 (540–660)	660 (540–660)
Total Sleep	26	0.90 (0.78–0.95)	810 (750–810)	810 (750–870)

* *N* = 28 parents (Group 1: MBQ-B Open on Day 7 and Day 10). *N* = 6 had not reached the rolling milestone and completed the tummy time item. Deletions for missing data on Day 7 or Day 10 were as follows: Active Play (*N* = 2); Restrained Time (*N* = 2); Non-interactive Screen Time (*N* = 1), Interactive Screen Time (*N* = 1); Sleep (*N* = 1)

* *N* = 28 parents (Group 2: MBQ-B Closed on Day 7 and Day 10). *N* = 2 had not reached the rolling milestone and completed the tummy time item. Deletions for missing data on Day 7 or Day 10 were as follows: Active Play (*N* = 2); Restrained Time (*N* = 2); Non-interactive Screen Time (*N* = 2), Interactive Screen Time (*N* = 2); Sleep (*N* = 2)

Table 3 reports the validity results for the open- and closed-ended versions of the MBQ-B. For the open-ended version, strong positive correlations were observed between the 24-hour TUD recorded and MBQ-B reported tummy time, non-interactive screen time, total screen time, and daytime sleep. Moderate positive correlations were observed for active play, interactive screen time, and total sleep duration. Weak associations were observed for restrained time and night-time sleep duration; however, the positive correlation between 24-hour TUD recorded and MBQ-B reported night-time sleep was statistically significant. For the closed-ended version of the MBQ-B, strong positive correlations were observed for non-interactive screen time and total screen time, while the positive correlations for tummy time, interactive screen time, and night-time sleep were moderate in strength. Statistically significant positive associations were observed between 24-hour TUD recorded and MBQ-B reported daytime sleep and total sleep duration; however, the correlations were just below the moderate threshold (rho = 0.37).

**Table 3:** Validity coefficients and median duration for movement behaviours measured by the 24-hour time use diary (TUD) and the open- or closed-ended versions of the MBQ-B

MBQ-B Open				
		Validity	Median (25th– 75th percentile)	
	N	**Spearman rho**	**MBQ-B (mins/day)**	**24-h TUD (mins/day)**
**Physical Activity**				
Tummy Time	8	0.77 *	25 (13–35)	11.3 (0–52)
Active Play	30	0.63 **	300 (210–420)	270 (188–371)
Restrained Time	36	0.22	147 (90–210)	165 (112–221)
**Screen Time**				
Non-interactive	38	0.84 ***	7.5 (0–30)	0 (0–23)
Interactive	38	0.53 **	0 (0–15)	0 (0–4)
Total Screen	38	0.87 ***	15 (0–38)	7.5 (0–30)
**Sleep**				
Day Sleep	37	0.70 ***	155 (120–210)	165 (128–203)
Night Sleep	37	0.34 *	660 (600–690)	675 (608–713)
Total Sleep	37	0.55 **	810 (758–870)	836 (746–893)
**MBQ-B Closed**				
**Physical Activity**				
Tummy Time	4	0.54	7.5 (5–15)	22.5 (0–45)
Active Play	29	0.68 ***	105 (75–120)	210 (143–300)
Restrained Time	33	0.48 *	105 (75–105)	150 (90–188)
**Screen Time**				
Non-interactive	33	0.76 ***	7.5 (0–22.5)	0 (0–15)
Interactive	33	0.51 **	0 (0–7.5)	0 (0–0)
Total Screen	33	0.77 ***	7.5 (0–22.5)	7.5 (0–15)
**Sleep**				
Day Sleep	33	0.37 *	150 (150–150)	158 (135–195)
Night Sleep	33	0.47 **	660 (540–660)	675 (630–720)
Total Sleep	33	0.37 *	810 (690–810)	840 (788–878)

*** *P* < 0.0001, ** *P* < 0.001, * *P* < 0.05

*N* = 43 parents (*N* = 28 Group 1 and *N* = 15 Group 3; MBQ-B Open on Day 7). *N* = 9 of the 43 babies had not reached the rolling milestone and completed the tummy time item. Deletions for missing data on the MBQ-B or 24-h TUD were as follows: tummy time (*N* = 1), Active Play (*N* = 4), Restrained Time (*N* = 7), Screen Time (*N* = 5), and Sleep (*N* = 6)

*N* = 42 parents (*N* = 28 Group 2 and *N* = 14 Group 4; MBQ-B Closed on Day 7). *N* = 5 of the 43 babies had not reached the rolling milestone and completed the tummy time item. Deletions for missing data on the MBQ-B or 24-h TUD were as follows: tummy time (*N* = 1), Active Play (*N* = 8), Restrained Time (*N* = 9), Screen Time (*N* = 9), and Sleep (*N* = 9)

Table 4 reports associations and the level of agreement between the movement behaviours measured by the open and closed-ended versions of the MBQ-B. Moderate to strong positive correlations were observed for all movement behaviours, with the exception of restrained time. Agreement for active play, non-interactive screen time, total screen time, and sleep duration ranged from moderate to excellent; however, agreement for restrained time and interactive screen time were poor. The relationship between the open and closed-ended versions of the tummy time item was not evaluated because only four parent-child dyads in Group 3 provided complete data on Day 7 and Day 10.

**Table 4:** Agreement and median duration of movement behaviours measured by the open- and closed-ended versions of the MBQ-B

		Agreement		Median (25th– 75th percentile)	
		Spearman Rho	ICC (95% CI)	Open (mins/day)	Closed (mins/day)
**Physical Activity**					
Tummy Time	6	0.89 **	0.76 (0.34–0.95)	20.5 (8–25)	22.5 (2.5–35)
Active Play	14	0.77 **	0.67 (0.37–0.88)	180 (160–210)	120 (105–120)
Restrained Time	20	0.79 ***	0.32 (0.07–0.73)	150 (90–170)	113 (75–120)
**Screen Time**					
Non-interactive	23	0.91 ***	0.57 (0.29–0.82)	4 (0–15)	7.5 (0–7.5)
Interactive	23	0.39	0.33 (0.06–0.79)	0 (0–0)	0 (0–7.5)
Total Screen	23	0.91 ***	0.62 (0.33–0.84)	9 (0–15)	7.5 (0–15)
**Sleep**					
Day Sleep	23	0.89 ***	0.88 (0.74–0.95)	173 (150–210)	150 (150–210)
Night Sleep	23	0.69 **	0.53 (0.25–0.79)	630 (600–660)	660 (540–660)
Total Sleep	23	0.70 **	0.73 (0.47– 0.89)	780 (758–840)	810 (750–810)

*** *P* < 0.0001, ** *P* < 0.001, * *P* < 0.05

*N* = 29 parents (*N* = 15 Group 3 and *N* = 14 Group 4; MBQ-B Open or the MBQ-B Closed on Day 7 and Day 10). *N* = 6 babies (*N* = 3 Group 3, *N* = 3 Group 4) had not reached the rolling milestone and completed the tummy time item. Deletions due to missing data on either the open or closed versions of the MBQ-B were as follows: Active Play (*N* = 9, Group 3: *N* = 5, Group 4: *N* = 4); Restrained Time (*N* = 9, Group 3: *N* = 5, Group 4: *N* = 4); Screen Time (*N* = 6, Group 3: *N* = 2 Group 4: *N* = 4); Sleep (*N* = 6, Group 3: *N* = 2 Group 4: *N* = 4)

### Results for the MBQ-C

Of the 215 parent-child dyads, 198 (92%) completed at least one MBQ-C questionnaire, with 161 parents (75%) completing the two MBQ-C questionnaires as instructed on Day 7 and Day 10, respectively. Of the 198 parents completing one MBQ-C, 184 (86%) completed the 24-hour TUD. Of the 215 children sent an accelerometer pack, 15 refused to wear the monitor, while an additional 51 children did not achieve the required four valid monitoring days. In total, 145 parent-child dyads (67%) completed at least one MBQ-C questionnaire, completed the 24-hour TUD, and provided valid accelerometer data. Compared to those completing all planned assessments, parents with missing data on at least one measure tended to be younger, reside in a postcode with a lower ISRAD, more likely to have a single child under the age of five, and less likely to have their child attend childcare. However, there were no meaningful differences in the percentage of parents with a university degree, child sex, child age, and child weight status (see supplementary material 1).

Table 5 reports the test-retest reliability results for the open-and closed-ended version of the MBQ-C. ICCs for the open-ended version of the MBQ-C were moderate to excellent with ICC’s ranging from 0.68 to 0.95, with 10 of the 12 ICCs exceeding 0.80. ICCs for the closed-ended version of the MBQ-C were moderate to excellent with ICCs ranging from 0.58 to 0.95. Compared to the open-ended version, ICCs for the closed-ended version were lower for non-interactive sedentary screen time, total screen time, total sedentary screen time, day sleep, night sleep and total sleep duration, while higher for active play, energetic play, non-interactive screen time, and interactive screen time. Seven of the 12 ICCs for closed version exceeded 0.80.

**Table 5:** Test-retest reliability coefficients and median duration for movement behaviours reported on Day 7 and 10 for the open- and closed-ended versions of the MBQ-C

MBQ-C Open				
	N	**Reliability**	**Median (25th– 75th percentile)**	
	N	**ICC (95% CI)**	**Day 7 (mins/day)**	**Day 10 (mins/day)**
**Physical Activity**				
Active Play	46	0.80 (0.67–0.88)	240 (180–360)	240 (180–320)
Energetic Play	46	0.82 (0.71–0.90)	83 (60–124)	92 (60–120)
**Screen Time**				
Non-interactive	44	0.86 (0.75–0.92)	94 (47–137)	92 (47–137)
Interactive	44	0.72 (0.56–0.84)	6 (0–17)	9 (1–17)
Total Screen	44	0.88 (0.80–0.93)	99 (52–146)	108 (60–148)
**SED Screen Time**				
Non-interactive	44	0.83 (0.72–0.90)	63 (43–106)	66 (34–109)
Interactive	44	0.68 (0.48–0.80)	0 (0–15)	0 (0–15)
Total SED Screen	44	0.86 (0.77–0.92)	73 (47–111)	75 (35–120)
**Sleep**				
Day Sleep	46	0.98 (0.96–0.99)	0 (0–90)	0 (0–90)
Night Sleep	46	0.84 (0.74–0.91)	630 (600–660)	625 (570–660)
Total Sleep	46	0.85 (0.75–0.91)	660 (620–720)	660 (630–720)
Sleep Routine	46	0.95 (0.92–0.97)	7 (6–7)	7 (6–7)
**MBQ-C Closed**				
**Physical Activity**				
Active Play	54	0.88 (0.80–0.93)	193 (133–214)	193 (107–219)
Energetic Play	54	0.86 (0.77–0.91)	75 (39–105)	75 (45–96)
**Screen Time**				
Non-interactive	54	0.90 (0.84–0.94)	54 (24–96)	54 (24–105)
Interactive	54	0.67 (0.59–0.80)	4 (0–15)	12 (0–15)
Total Screen	54	0.85 (0.76–0.91)	64 (30–111)	69 (39–111)
**SED Screen Time**				
Non-interactive	54	0.79 (0.66–0.88)	39 (15–66)	41 (17–75)
Interactive	54	0.77 (0.62–0.86)	4 (0–11)	7 (0–15)
Total SED Screen	54	0.82 (0.77–0.92)	39 (20–80)	41 (30–83)
**Sleep**				
Day Sleep	54	0.90 (0.82–0.94)	30 (0–90)	30 (0–90)
Night Sleep	54	0.58 (0.36–0.73)	660 (540–600)	660 (540–600)
Total Sleep	54	0.71 (0.56–0.82)	660 (630–690)	660 (570–750)
Sleep Routine	54	0.95 (0.92–0.97)	7 (6–7)	7 (6–7)

*N* = 72 parents (Group 1: MBQ-C Open on Day 7 and Day 10). Deletions for missing data on Day 7 or 10 were as follows: Physical Activity (*N* = 26), Non-interactive Screen Time (*N* = 28), Interactive Screen Time (*N* = 28), Sleep (*N* = 26)

*N* = 72 parents (Group 2: MBQ-C Closed on Day 7 and Day 10). Deletions for missing data on Day 7 or 10 were as follows: Physical Activity (*N* = 18), Non-interactive Screen Time (*N* = 18), Interactive Screen Time (*N* = 28), Sleep (*N* = 18)

Table 6 reports the validity results for the screen time and sleep items on the open- and closed-ended versions of the MBQ-C. For the open-ended version, strong positive associations were observed between the 24-hour TUD recorded and MBQ-C reported non-interactive screen time, total screen time, total sedentary screen time, daytime sleep, and total sleep duration. Moderate positive associations were observed for the remaining screen time and sleep variables, with correlations ranging from 0.47 to 0.67. For the closed-ended version of the MBQ-C, strong positive correlations were observed for total screen time, total sedentary screen time, and daytime sleep duration. Moderate positive associations were observed for the remaining screen time and sleep variables, with Spearman correlations ranging from 0.44 to 0.67.

**Table 6:** Validity coefficients and median durations for movement behaviours measured by the 24-hour time use diary (TUD) and the open- or closed-ended versions of the MBQ-C

MBQ-C Open				
		Validity	Median (25th– 75th percentile)	
	N	Spearman rho ***	**MBQ-C (mins/day)**	**24-h TUD (mins/day)**
**Screen Time**				
Non-interactive	75	0.72	81.4 (38.6–128.6)	67.5 (15.0–120.0)
Interactive	75	0.49	5.7 (0–17.1)	0 (0–15)
Total Screen	75	0.79	92.1 (45.0–137.1)	90.0 (30.0–150.0)
**SED Screen Time**				
Non-interactive	75	0.67	60.0 (30.0–98.3)	52.5 (15.0–97.5)
Interactive	75	0.47	0 (0–12.9)	0 (0–7.5)
Total SED Screen	75	0.75	64.3 (32.9–107.1)	67.5 (22.5–112.5)
**Sleep**				
Day Sleep	70	0.83	25.0 (0–97.5)	37.5 (0–90)
Night Sleep	70	0.62	600 (600–660)	638 (593–668)
Total Sleep	70	0.70	660 (620–720)	683 (645–720)
**MBQ-C Closed**				
**Screen Time**				
Non-interactive	91	0.66	53.6 (23.6–105.0)	60.0 (22.5–131.3)
Interactive	90	0.51	4.3 (0–15.0)	0 (0–15)
Total Screen	91	0.71	68.6 (32.1–128.6)	78.8 (30.0–142.5)
**SED Screen Time**				
Non-interactive	91	0.63	41.8 (18.2–76.1)	52.5 (15.0–123.8)
Interactive	90	0.51	4.3 (0–11.8)	0 (0–7.5)
Total SED Screen	91	0.70	49.7 (22.5–88.9)	60 (15.0–138.8)
**Sleep**				
Day Sleep	77	0.86	30 (0–90)	18.8 (0–97.5)
Night Sleep	77	0.44	660 (540–660)	638 (600–660)
Total Sleep	77	0.58	660 (630–690)	668 (623–735)

*** *P* < 0.0001, ** *P* < 0.001, * *P* < 0.05

*N* = 107 (*N* = 72 Group 1 and *N* = 35 Group 3: MBQ-C Open on Day 7). Deletions due to missing data on the MBQ-C or 24-h TUD were as follows: Non-interactive Screen Time (*N* = 32), Interactive Screen time (*N* = 32), and Sleep (*N* = 37)

*N* = 108 (*N* = 72 Group 2 and *N* = 36 Group 4: MBQ-C Closed on Day 7). Deletions due to missing data on the MBQ-C or 24-h TUD were as follows: Non-interactive Screen Time (*N* = 17) and Interactive Screen time (*N* = 18), and Sleep (*N* = 31)

Associations between MBQ-C reported physical activity and device-measured physical activity are summarised in Table 7. For both the open- and closed-ended versions, parent/caregiver reported time in active play was positively associated with average wrist acceleration and device-measured total physical activity (TPA). Similarly, parent/caregiver reported time in MVPA was positively and significantly associated with average wrist acceleration and device-measured MVPA. Correlations were statistically significant but weak in magnitude (rho = 0.25–0.39).

**Table 7:** Associations between MBQ-C reported physical activity and device-measured physical activity

	Mean Acceleration		Device-measured Total PA		Device-measured MVPA
MBQ-C Open (**N = 61**)	Spearman rho				
Active Play	0.33 *		0.25 *		0.37 *
Energetic Play	0.36 *		0.20		0.39 *
**MBQ-C Closed** (**N = 66**)					
Active Play	0.30 *		0.27 *		0.30 *
Energetic Play	0.32 *		0.23		0.35 *
**Descriptive Statistics - Median** (25^th^– 75^th^ percentile)					
		**MBQ-C Open** (**N = 61**)		**MBQ-C Closed** (**N = 66**)	
MBQ-C Active Play (min/day)		240 (180–300)		167 (120–257)	
MBQ-C Energetic Play (min/day)		94 (60–163)		64 (39–105)	
Mean Acceleration (m*g*)		42.7 (33.3–48.7)		42.3 (34.2–50.9)	
Device Measured Total PA (min/day)		256 (220–295)		258 (225–293)	
Device Measured MVPA (min/day)		40 (29–60)		44 (34–51)	

*N* = 107 (*N* = 72 Group 1 and *N* = 35 Group 3) parent-dyads were sent an accelerometer (Day 0) and completed the MBQ-C Open on Day 7. Of the 107, *N* = 17 had missing data for the accelerometer assessment and the MBQ-C; *N* = 13 had missing or non-valid accelerometer data; *N* = 16 had missing or non-valid MBQ-C responses

*N* = 108 (*N* = 72 Group 2 and *N* = 36 Group 4) parent-child dyads were sent an accelerometer (Day 0) and completed the MBQ-C Closed on Day 7. Of the 108, *N* = 11 had missing data for the accelerometer assessment and the MBQ-C; *N* = 25 had missing or non-valid accelerometer data; *N* = 16 had missing or non-valid MBQ-C responses

Associations and the level of agreement between the movement behaviours measured by the open and closed-ended version of the MBQ-C are reported in Table 8. Moderate to strong positive correlations were observed for all movement behaviours, while the corresponding ICCs ranged from moderate to excellent.

**Table 8:** Agreement and median durations of movement behaviours measured by the open- and closed-ended versions of the MBQ-C

		Agreement		Median (25th– 75th percentile)	
	N	**Spearman rho**	**ICC (95% CI)**	**Open (mins/day)**	**Closed (mins/day)**
**Physical Activity**					
Active Play	49	0.57 ***	0.50 (0.24–0.66)	197.1 (154.2–300)	167.1 (132.9–210.0)
Energetic Play	49	0.56 ***	0.50 (0.23–0.68)	94.3 (60–120)	66.4 (45.0–98.6)
**Screen Time**					
Non-interactive	50	0.89 ***	0.80 (0.68–0.88)	77.1 (40.0–120.0)	49.3 (23.6–105.0)
Interactive	51	0.74 ***	0.67 (0.51–0.80)	7.2 (0–15)	4.3 (0–15)
Total Screen	51	0.91 ***	0.81 (0.70–0.89)	85.7 (46.1–122.1)	55.7 (26.8–107.1)
**SED Screen Time**					
Non-interactive	50	0.89 ***	0.82 (0.72–0.90)	60 (31.4–96.4)	43.9 (16.1–88.9)
Interactive	51	0.67 ***	0.80 (0.68–0.88)	0 (0–10.7)	2.1 (0–7.5)
Total SED Screen	51	0.94 ***	0.88 (0.80–0.93)	60 (33.6–104.6)	45 (21.9–92.7)
**Sleep**					
Day Sleep	50	0.92 ***	0.82 (0.72–0.89)	0 (0–105)	30 (0–90)
Night Sleep	50	0.78 ***	0.90 (0.84–0.94)	600 (600–660)	660 (540–660)
Total Sleep	50	0.75 ***	0.83 (0.73–0.90)	660 (615–710)	660 (630–690)
Sleep Routine	50	0.81 ***	0.76 (0.61–0.86)	7 (6–7)	7 (6–7)

*N* = 71 parents (*N* = 35 Group 3 and *N* = 36 Group 4; MBQ-C Open or the MBQ-C Closed on Day 7 and Day 10). Deletions due to missing data on the open or closed versions of the MBQ-C were as follows: Active Play and Energetic Play (*N* = 22, Group 3: *N* = 14 Group 4: *N* = 8); Non-interactive Screen Time (*N* = 21, Group 3: *N* = 11 Group 4: *N* = 10); Interactive Screen Time (*N* = 20, Group 3: *N* = 12 Group 4: *N* = 8); Sleep (*N* = 21, Group 3: *N* = 11 Group 4: *N* = 10)

## Discussion

The current study assessed the psychometric properties of two newly developed short-form questionnaires to assess 24-hour movement behaviours in children aged 0 to 5 years; the MBQ-B for infants and toddlers who have not yet reached their walking milestone, and the MBQ-C for toddlers and pre-schoolers who have achieved their walking milestone. Both questionnaires exhibited moderate-to-strong evidence of test-retest reliability and acceptable evidence of concurrent validity for the assessment of tummy time (in infants), physical activity, screen time, and sleep. Moreover, agreement between the open- and closed-ended versions of the MBQ-B and MBQ-C ranged from moderate to excellent, indicating that the two versions provided comparable information despite having different response formats.

To our knowledge, the MBQ-B and MBQ-C are the first validated proxy-report questionnaires specifically designed to rapidly assess 24-hour movement behaviours in young children aged 0 to 5 years. Informed by the results of an extensive cognitive interviewing study [33], the MBQ tools utilize a number of innovative design features to enhance the assessment of 24-hour movement behaviours in young children. First, items are based on developmental milestones rather than chronological age. This is an important design feature considering the wide confidence intervals around the age assigned to key developmental milestones such as rolling over (4 months) and walking (12 months). Hence, tummy time is assessed among infants who have not yet met their rolling over milestone, while floor-based active play is assessed among toddlers who have not yet reached the walking milestone. Second, for toddlers and pre-schoolers who have reached their walking milestone, the MBQ-C assesses compliance with contemporary physical activity recommendations by measuring total daily time engaged in active play and the amount of active play time in energetic or vigorous intensity physical activities. Third, the MBQ tools distinguishes interactive screen time such as doing puzzles, playing games, or video chatting from non-interactive or passive screen time such as watching television programs, videos/internet clips, or movies. Moreover, consistent with prevailing guidelines on screen use among young children, the MBQ-C differentiates sedentary or seated screen time from standing or active screen time. Fourth and finally, both the MBQ-B and MBQ-C can be administered using an open-ended or closed-ended response format, giving researchers and practitioners the flexibility to select a questionnaire and response format that best supports their use case. Collectively, these design features enable researchers and practitioners to efficiently evaluate compliance with 24-hour movement guidelines, as well as the individual recommendations related to physical activity, screen time, and sleep.

The reliability and validity coefficients observed for the MBQ-B and MBQ-C approximated or exceeded those reported in recent systematic reviews of studies measuring screen time in children aged 0 to 6 years [20] and the psychometric properties of proxy-report questionnaires assessing 24-hour movement behaviours in young children [18, 19]. Test-retest reliability for the physical activity, screen time, and sleep outcomes was moderate to excellent, with ICCs ranging from 0.71 to 0.94 and 0.58 to 0.98 for the MBQ-B and MBQ-C, respectively. Moreover, with the exception of restrained time (open-ended MBQ-B) and sleep duration (closed-ended MBQ-B), validity coefficients for total screen time and sleep duration were moderate to strong in magnitude, with Spearman correlations ranging from 0.55 to 0.87 and 0.58 to 0.79 for the MBQ-B and MBQ-C, respectively.

Test-retest reliability was generally higher for the MBQ-B outcomes compared to those measured by the MBQ-C. The open-ended version of the MBQ-B achieved good to excellent reliability on all nine outcomes measured, while the closed-ended version of the MBQ-B achieved good to excellent reliability on six of the nine outcomes measured. In comparison, test-retest reliability for the MBQ-C outcomes was less consistent and ranged from moderate to excellent. The factors contributing to the higher reliability of the MBQ-B outcomes are not clear. It is possible that infant movement behaviours were reported with greater consistency because they were more discrete in nature (i.e., tummy time, active floor play, restrained time vs. playing outside), completed as part of a daily routine, and required parental supervision or co-participation. Additionally, because fewer infants attend childcare on a regular basis, parents completing the MBQ-B may have been more aware of their child’s daily activities.

ICCs for the open-ended versions of the MBQ questionnaires tended to be more consistent and greater in magnitude than those measured by closed-ended versions Although the majority of outcomes measured by the open- or closed-ended versions of the MBQ-C achieved good test-retest reliability or better, the ICCs for interactive screen time and nighttime sleep duration (closed-ended version only) were below 0.70. The lower reliability observed for these outcomes may be attributable, at least in part, to floor effects and lack of variation in the parent responses to these items. Notably, the median time for interactive screen use ranged from 0 to 12 min per day; while for nighttime sleep duration, over 90% of parents completing the closed-ended version of the MBQ-C endorsed the “between 8 and 10 hours per night” or “between the 10 and 12 hours per night” response options on Day 7 and Day 10.

In absolute terms, the significant positive correlations between active play, as measured by the MBQ-C, and device-measured physical activity were weak in magnitude. However, compared to previous studies using accelerometers to validate parent-reports of physical activity in young children, the results are typical and supportive of our conclusion that the MBQ-C provides acceptably valid estimates of daily time in active play and MVPA. Burdette et al. [34] assessed the validity of two parent report physical activity measures in a sample of 250 preschool children - the outdoor playtime checklist and a 1-month outdoor playtime recall. For the checklist, the correlation between parent reported outdoor playtime and device-measured physical activity was 0.33. For the 1-month recall, the correlation with device-measured physical activity was just 0.20. In an evaluation of the parent-completed Preschool-aged Children’s Physical Activity Questionnaire (Pre-PAQ), Dwyer and colleagues [35] reported correlations between parent reported physical activity with device-measured physical activity ranging from − 0.07 to 0.28. Sarker and colleagues [36] assessed the association between parent-reported physical activity and device measured physical activity in Canadian children aged 6 years and under. The correlation between parent reported physical activity and device measured MVPA was 0.39. In an evaluation of the Early Years Physical Activity Questionnaire, Bingham et al. [37] reported a correlation of 0.30 between parent reported physical activity and device-measured MVPA. Most recently, Goncalves et al. [38] assessed the validity of the Burdette 1-month proxy recall in 78 parent-child dyads from rural Brazil. The correlation between parent-reported physical activity and device measured MVPA was 0.39.

The limitations of proxy-report physical activity questionnaires are well-documented [15, 16]. Unlike sleep and screen use, it is extremely difficult for parents to monitor their child’s movement behaviours across an entire day, particularly if their child spends significant amounts of time with other family members and/or attends childcare regularly. Moreover, the sporadic and pulsatile nature of young children’s physical activity makes it extremely difficult for parents to estimate the frequency, intensity, and duration of physical activity [25, 26]. These limitations were apparent in our cognitive interviewing study which utilised “think-out-loud” methods to understand how parents retrieved, encoded, and formulated responses to question about their child’s physical activity and sedentary behaviours [33]. When estimating the frequency and/or duration of active play, parents thought about their child’s daily routine, considering wake and bedtimes, daytime naps or eating occasions, and reported any time spent outdoors as active play, regardless of the actual intensity of activity. This observation supports the view that parent proxy reports of young children’s movement behaviours are subject to considerable recall bias and that, whenever possible, device-based measures of physical activity should be used. However, for monitoring and evaluation scenarios in which device-based measures are not feasible or unavailable, our results indicate that the MBQ tools can be used to obtain valid and reliable assessments of relative participation in developmentally-significant forms of physical activity in children aged five years and under.

The current study had several strengths. First, the items included in the MBQ tools were informed by extensive systematic reviews [17, 20] and a comprehensive cognitive interviewing study asking parents to review the format, content, and clarity of questionnaire items and response options [33]. Second, test-retest reliability and concurrent validity were assessed in a national sample comprising parent-child dyads from all states and territories across Australia, with representation from all ten deciles for relative social and economic advantage and disadvantage [32]. Third, device-measured total physical activity and daily MVPA estimates were generated using the latest machine learning accelerometer data processing methods which overcome the significant limitations of cut-point approaches in young children [25, 26].

Opposing these strengths were a number of limitations. First, given that the MBQ questionnaires were developed and tested in Australian parents and children, the results may not be generalisable to other nationalities and cultures. Second, a relatively small number of families had infants who had not reached their rolling milestone and completed the tummy time items. As such, the reported validity and test-retest reliability coefficients for the tummy-time items should be interpreted with considerable caution. Importantly, test-retest reliability for the closed-ended version of the tummy time item could not be estimated. Likewise, agreement between the open and closed-ended version of the tummy time question could be not be evaluated. Third and finally, a significant proportion of parents completing the MBQ-C had missing data due to non-response and/or insufficient accelerometer wear time. Consequently, the reliability and validity estimates for the MBQ-C may be subject to bias. Of the 215 parents completing the MBQ-C, 161 completed repeat questionnaires on Day 7 and Day 10. Of the 215 children sent an accelerometer pack, 15 refused to wear the monitor, while an additional 51 children did not provide the required four monitoring days with accelerometer wear time ≥ 960 min per day. These findings highlight the challenges and complexities of administering multiple online questionnaires, 24-hour time use dairies, and device-based physical activity measures in time-poor households over a 10-day period.

## Conclusion

In conclusion, the MBQ-B and MBQ-C are valid and reliable short form assessment tools for measuring 24-hour movement behaviours in infants, toddlers, and pre-schoolers. Both the open- and closed-ended versions of the MBQ-B and MBQ-C are suitable for research conducted for policy and practice purposes, including the evaluation of scaled-up early obesity prevention programs. Future studies should evaluate the psychometric properties of the MBQ-B and MBQ-C in population-based samples from other counties and cultures, including families residing in low-to-middle income countries. Furthermore, in order to evaluate the reliability and validity of the tummy time items more comprehensively, future studies should purposively oversample infants who have not reached their rolling milestone. Considering the modest validity observed for the MBQ items measuring restrained time in young children, future investigations should consider making refinements to these items and evaluating validity. Finally, future studies evaluating the MBQ-B and MBQ-C should examine additional psychometric properties such as responsiveness to change and the smallest detectable change for the MBQ outcomes.

### Electronic supplementary material

Below is the link to the electronic supplementary material.

Supplementary Material 1

Supplementary Material 2

Supplementary Material 3

## Data Availability

The dataset supporting the conclusions of this article can be made available upon request after approval by the authors. Please direct inquiries to s.trost@uq.edu.au.

## Abbreviations

MBQ-B: Movement Behaviour Questionnaire Baby
MBQ-C: Movement Behaviour Questionnaire Child
TUD: Time Use Diary
ICC: Intraclass Correlation Coefficient
ENMO: Euclidian Norm Minus One gravitational unit
MVPA: Moderate-to-Vigorous intensity Physical Activity
ISRAD: Index of Relative Socio-economic Advantage and Disadvantage
