# Supplementary material for: Reliability and validity of rapid assessment tools for measuring 24-hour movement behaviours in children aged 0–5 years: the Movement Behaviour Questionnaire Baby (MBQ-B) and child (MBQ-C)
Source: Int J Behav Nutr Phys Act. 2024 Apr 23;21:43. doi: 10.1186/s12966-024-01596-5 (PMC11041005; doi:10.1186/s12966-024-01596-5)
Supplement: Supplementary file 1 — Supplementary Material 1 [file 12966_2024_1596_MOESM1_ESM.docx]

Supplementary Table 1. Descriptive characteristics of parents and children in the MBQ-C sample completing all assessments and those with missing data on one or more assessments.

|  | **Total Sample (N = 215)** | **Non-missing data on all assessments**  **(N = 125)** | **Missing data on ≥ 1 assessments**  **(N = 90)** |
| --- | --- | --- | --- |
| **Parent Respondent Age (years)** |  |  |  |
| 18 – 25 | 2.3% | 0.0% | 5.6% |
| 26 – 35 | 51.2% | 48.0% | 55.6% |
| 36 – 45 | 44.7% | 50.4% | 36.7% |
| > 45 | 1.9% | 1.6% | 2.2% |
| **Parent Respondent Education** |  |  |  |
| High School Certificate | 7.0% | 5.6% | 8.9% |
| TAFE/Diploma/Certificate | 19.5% | 21.6% | 16.7% |
| University Undergraduate | 35.8% | 35.2% | 36.7% |
| University Postgraduate | 37.7% | 37.6% | 37.8% |
| **SEIFA Decile (IRSAD)** |  |  |  |
| 1 – 2 (most disadvantaged) | 8.9% | 4.8% | 14.4% |
| 3 - 4 | 12.4% | 14.4% | 10.0% |
| 5 - 6 | 22.8% | 24.0% | 21.1% |
| 7 - 8 | 29.3% | 31.2% | 26.7% |
| 9 – 10 (least disadvantaged) | 26.6% | 25.6% | 27.8% |
| **Child Sex** |  |  |  |
| Female | 45.6% | 48.0% | 42.2% |
| Male | 54.4% | 52.0% | 57.8% |
| **Child Age (months)** | 37.8 ± 14.8 | 39.9 ± 15.1 | 35.0 ± 13.9 |
| **Child Care Attendance** |  |  |  |
| 0 days | 16.7% | 12.0% | 23.3% |
| 1 – 3 days/week | 44.2% | 47.2% | 40.0% |
| 4 – 5 days/week | 39.1% | 40.8% | 36.7% |
| **Number of children in household aged 5 years or younger** |  |  |  |
| 1 Child | 52.1% | 51.1% | 82.2% |
| 2 Children | 43.3% | 44.4% | 6.7% |
| ≥ 3 Children | 4.6% | 4.4% | 11.1% |
| **Child BMI z-score** | 0.64 ± 1.7 | 0.53 ± 1.8 | 0.80 ± 1.7 |
| **Child Weight Status *** |  |  |  |
| Underweight | 5.8% | 7.1% | 4.0% |
| Healthy weight | 54.0% | 53.1% | 55.3% |
| At-risk of overweight | 21.2% | 22.1% | 19.7% |
| Overweight | 11.6% | 11.5% | 11.8% |
| Obese | 7.4% | 6.2% | 9.2% |

IRSAD = Index of Relative Socio-economic Advantage and Disadvantage

* WHO Child Growth Standards for Children 0 – 5 Years based on parent-reported height and weight.

Supplementary Table 2. Descriptive characteristics of parents and children in the MBQ-B sample completing all assessments and those with missing data on one or more assessments.

|  | **MBQ-B Sample (N=85)** | **Non-missing data on all assessments**  **(N = 70)** | **Missing data on ≥ 1 assessments**  **(N = 15)** |
| --- | --- | --- | --- |
| **Parent Respondent Age (years)** |  |  |  |
| 18 – 25 | 4.7% | 2.9% | 13.3% |
| 26 – 35 | 68.2% | 72.9% | 46.7% |
| 36 – 45 | 27.1% | 24.3% | 40.0% |
| > 45 | 0.0% | 0.0% | 0.0% |
| **Parent Respondent Education** |  |  |  |
| High School Certificate | 11.8% | 8.6% | 26.7% |
| TAFE/Diploma/Certificate | 21.2% | 17.1% | 40.0% |
| University Undergraduate | 35.3% | 41.4% | 6.7% |
| University Postgraduate | 31.8% | 32.9% | 26.7% |
| **SEIFA Decile (IRSAD)** |  |  |  |
| 1 – 2 (most disadvantaged) | 8.2% | 10.0% | 0.0% |
| 3 - 4 | 20.0% | 17.2% | 33.3% |
| 5 - 6 | 21.0% | 24.3% | 40.0% |
| 7 - 8 | 22.4% | 21.4% | 26.7% |
| 9 – 10 (least disadvantaged) | 22.4% | 27.2% | 0.0% |
| **Child Sex** |  |  |  |
| Female | 49.4% | 48.6% | 53.3% |
| Male | 50.6% | 51.4% | 46.7% |
| **Child Age (months)** | 6.5 ± 3.4 | 7.8 ± 3.4 | 6.3 ± 3.3 |
| **Child Care Attendance** |  |  |  |
| 0 days | 45.9% | 44.3% | 53.3% |
| 1 – 3 days/week | 36.5% | 40.0% | 20.0% |
| 4 – 5 days/week | 17.6% | 15.7% | 26.7% |
| **Number of children in household aged 5 years or younger** |  |  |  |
| 1 Child | 62.4% | 55.7% | 93.3% |
| 2 Children | 34.1% | 41.4% | 6.7% |
| ≥ 3 Children | 3.5% | 2.9% | 0.0% |
| **Child BMI z-score** | 0.30 ± 1.6 | 0.41 ± 1.6 | -0.17 ± 1.6 |
| **Child Weight Status *** |  |  |  |
| Underweight | 9.3% | 8.1% | 15.4% |
| Healthy weight | 58.7% | 56.5% | 69.2% |
| At-risk of overweight | 18.7% | 22.6% | 0.0% |
| Overweight | 8.0% | 8.1% | 7.7% |
| Obese | 5.3% | 4.8% | 7.7% |

IRSAD = Index of Relative Socio-economic Advantage and Disadvantage

* WHO Child Growth Standards for Children 0 – 5 Years based on parent reported height and weight.
