# Supplementary material for: Reliability and validity of rapid assessment tools for measuring 24-hour movement behaviours in children aged 0–5 years: the Movement Behaviour Questionnaire Baby (MBQ-B) and child (MBQ-C)
Source: Int J Behav Nutr Phys Act. 2024 Apr 23;21:43. doi: 10.1186/s12966-024-01596-5 (PMC11041005; doi:10.1186/s12966-024-01596-5)
Supplement: Supplementary file 2 — Supplementary Material 2 [file 12966_2024_1596_MOESM2_ESM.pdf]

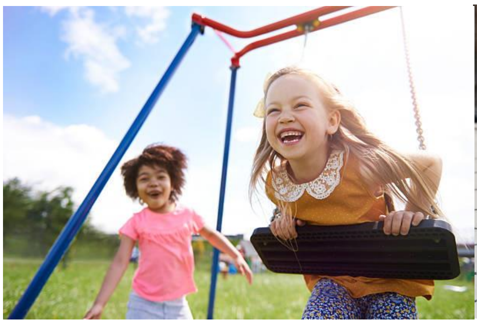
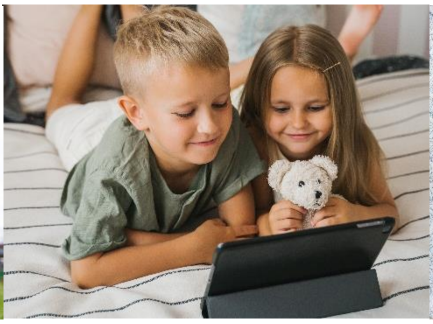
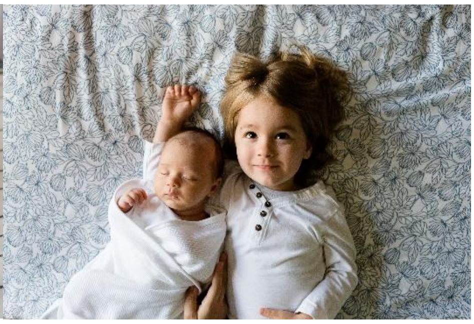

# Movement Behaviour Questionnaire

# Baby Open Version

# (MBQ-B)

OVERVIEW

- The MBQ is a validated rapid assessment tool for measuring movement behaviours in children aged 0 – 5 years.
- The MBQ-B measures tummy time or active play, restrained time, screen time and sleep.

TARGET POPULATION

For babies from 0 years who are
*yet to reach their walking milestone.*

HOW IT IS MEASURED

- 6 items
- Open-ended questions
- Self-report
- Hard-copy or electronic completion (REDCap data dictionary available upon request)
- Supplemented by the User guide for administration and scoring (hard copy or REDCap versions available)

Stewart. G. Trost

Contact for information: s.trost@uq.edu.au

Date: ____ / ____ / ______

# Movement Behaviour Questionnaire (MBQ-B)

## DIRECTIONS

This survey will ask you questions about your baby's movement behaviours (activity, screen time, and sleep) on a typical day.

A typical day is a day when your baby does things they normally do.

For questions about how much time your baby spends in these behaviours, please provide an answer to both hours and minutes, e.g., 2 hours 0 minutes, 0 hours 30 minutes.

Please respond to all the questions as best as you can.

---

How old is your baby?

- ◯ 0 – 3 months [1]
- ◯ 4 – 11 months [2]
- ◯ 12 months or older [3]

## This section is about your baby's tummy time and active play

Does your baby roll?

- ◯ Yes [1] – skip Q1A and Q1B and answer question Q2.
- ◯ No [0] – answer Q1A and Q1B and skip Q2.

**Q1A.** This question is about the times when your baby is awake and placed on their tummy for playtime while you are watching them.

Thinking about the past week, how many times EACH DAY did you usually place your baby on their tummy for play?

[ ] times

**Q1B.** How long did each tummy time usually last?

[ ] minutes

---

**Q2.** Thinking about the past week, on a TYPICAL DAY, how much time in total did you do some active play with your baby? *Active play could be crawling on the floor with your baby, rolling around the floor with your baby, playing at the park, dancing with your baby, chasing your baby.*

[ ] hours [ ] minutes

## This section is about your baby's restrained time (e.g., pram/stroller, highchair, or baby carrier)

**Q3A.** Thinking about the past week, on a TYPICAL DAY, how many times did you place your baby in a baby carrier or sling, car seat or capsule, stroller or pram, highchair, bouncer, jolly jumper or play pen?

[ ] times

**Q3B.** When your baby was in one of those devices, how long were they usually in it?

[ ] hours [ ] minutes

## This section is about your baby's screen time

**Q4.** Thinking about the past week, on a TYPICAL DAY, how much time did your baby spend watching television programs, videos/internet clips or movies on a television, computer or portable/mobile device such as iPad, tablet or smartphone?

[ ] hours [ ] minutes

**Q5** Thinking about the past week, on a TYPICAL DAY, how much time did your baby spend playing games, looking at photos, or video chatting (e.g. FaceTime, Zoom, Skype) on a screen-based device such as a computer or laptop, video game console, iPad, tablet, or smartphone?

[ ] hours [ ] minutes

## This section is about your baby's sleep

**Q6.** Thinking about the past week, on a TYPICAL NIGHT, how much time did your baby sleep in total during the night?

[ ] hours [ ] minutes

**Q7.** Thinking about the past week, on a TYPICAL DAY, how much time did your baby sleep in total during the day?

[ ] hours [ ] minutes

--- Thank you for completing the MBQ ---

# Calculation of outcome variables

| Outcome variable | Calculation |
|---|---|
| **TUMMY TIME** | |
| Total tummy time (minutes/day) | ______times (Q1A) x ______minutes per time (Q1B) |
| **ACTIVE PLAY** | |
| Total active play (minutes/day) | Q2 (______hours x 60) + ______minutes |
| **RESTRAINED TIME** | |
| Usual restrained time (minutes/bout) | Q3B (______hours x 60) + ______minutes |
| Total restrained time (minutes/day) | ______times (Q3A) x usual restrained time (Q3B) |
| **SCREEN TIME** | |
| Non-interactive screen time (minutes/day) | Q4 (______hours x 60) + ______minutes |
| Interactive screen time (minutes/day) | Q5 (______hours x 60) + ______minutes |
| Total screen time (minutes/day) | Non-interactive screen time + Interactive screen time |
| **SLEEP** | |
| Night sleep (minutes) | Q6 (______hours x 60) + ______minutes |
| Day sleep (minutes) | Q7 (______hours x 60) + ______minutes |
| Total sleep (minutes per 24 hours) | Night Sleep + Day Sleep |

# Truncation of extreme values

Implausible or extreme values for the following outcome variables are truncated (that is recoded) to the value equivalent of the 95th percentile from the validation study dataset.

| Outcome variable | Truncated value |
|---|---|
| Total tummy time | 180 minutes per day |
| Total active play | 480 minutes per day |
| Total restrained time | 360 minutes per day |

# Adherence to Guidelines

## Physical Activity

Tummy time (for babies who are yet to roll) – at least 30 minutes of tummy time per day.

Active Play (for babies who can roll) – at least 30 minutes of active play per day.

## Restrained Time

Not more than 60 minutes (1 hour) at a time of restrained time (such as in a stroller, car seat or highchair).

## Screen time

No screen time for babies/children under 2 years.

## Sleep

Age = 0 – 3 months: At least 840 minutes (14 hours) per day.

Age = 4 – 11 months: At least 720 minutes (12 hours) per day.

Age = 12 months or older: At least 660 minutes (11 hours) per day.

## <u>24-hour movement guidelines</u>

**For babies who are yet to roll:** meets all 3 guidelines for tummy time, screen time and sleep.

**For babies who can roll** meets all 3 guidelines for active play, screen time and sleep.

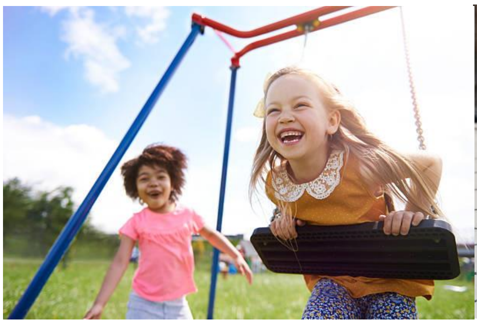
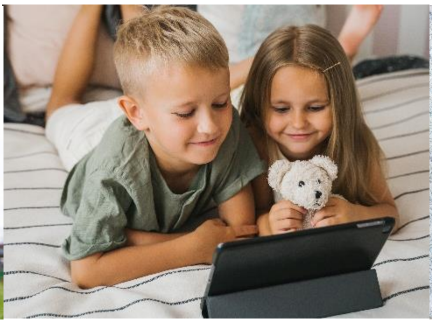
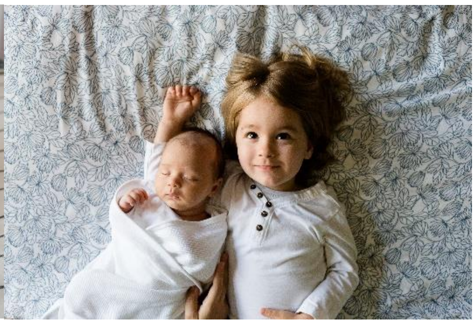

# Movement Behaviour Questionnaire

# Baby Closed Version

# (MBQ-B)

## OVERVIEW

- The MBQ is a validated rapid assessment tool for measuring movement behaviours in children aged 0 – 5 years.
- The MBQ-B measures tummy time or active play, restrained time, screen time and sleep.

## TARGET POPULATION

For babies from 0 years who are *yet to reach their walking milestone.*

## HOW IT IS MEASURED

- 6 items
- Closed questions
- Self-report
- Hard-copy or electronic completion (REDCap data dictionary available upon request)
- Supplemented by the User guide for administration and scoring (hard copy or REDCap versions available)

Stewart. G. Trost

Contact for information: s.trost@uq.edu.au

Date: ____ / ____ / ______

# Movement Behaviour Questionnaire (MBQ-B)

## DIRECTIONS

This survey will ask you questions about your baby's movement behaviours (activity, screen time, and sleep) on a typical day.

A typical day is a day when your baby does things they normally do.

Please respond to all the questions as best as you can.

---

How old is your baby?

- ◯ 0 – 3 months [1]
- ◯ 4 – 11 months [2]
- ◯ 12 months or older [3]

This section is about your baby's tummy time and active play

Does your baby roll?

- ◯ Yes [1] – skip Q1A and Q1B and answer question Q2.
- ◯ No [0] – answer Q1A and Q1B and skip Q2.

**Q1A.** This question is about the times when your baby is awake and placed on their tummy for playtime while you are watching them.

Thinking about the past week, how many times EACH DAY did you usually place your baby on their tummy for play?

- ◯ 0
- ◯ 1
- ◯ 2
- ◯ 3
- ◯ 4
- ◯ 5
- ◯ 6
- ◯ 7
- ◯ 8
- ◯ 9
- ◯ 10

**Q1B.** How long did each tummy time usually last?

- ◯ Less than 5 min [1]
- ◯ 5 – 10 min [2]
- ◯ 11 – 15 min [3]
- ◯ 16 – 20 min [4]
- ◯ 21 – 25 min [5]
- ◯ 26 – 30 min [6]
- ◯ More than 30 min [7]

**Q2.** Thinking about the past week, on a TYPICAL DAY, how much time in total did you do some active play with your baby? *Active play could be crawling on the floor with your baby, rolling around the floor with your baby, playing at the park, dancing with your baby, chasing your baby.*

◯ 0 min per day [0]
◯ Between 1 and 15 min per day [1]
◯ Between 15 and 30 min per day [2]
◯ Between 30 and 60 min per day [3]
◯ Between 1 and 1½ hrs per day [4]
◯ Between 1½ and 2 hrs per day [5]
◯ More than 2 hrs per day [6]

This section is about your baby's restrained time (e.g., pram/stroller, highchair, or baby carrier)

**Q3A.** Thinking about the past week, on a TYPICAL DAY, how many times did you place your baby in a baby carrier or sling, car seat or capsule, stroller or pram, highchair, bouncer, jolly jumper or play pen?

◯ 0
◯ 1
◯ 2
◯ 3
◯ 4
◯ 5
◯ 6
◯ 7
◯ 8
◯ 9
◯ 10

**Q3B.** When your baby was in one of those devices, how long were they usually in it?

◯ Less than 15 min per day [1]
◯ Between 15 and 30 min per day [2]
◯ Between 30 and 45 min per day [3]
◯ Between 45 and 60 min per day [4]
◯ Between 1 and 1½ hrs per day [5]
◯ Between 1½ and 2 hrs per day [6]
◯ More than 2 hrs per day [7]

## This section is about your baby's screen time

**Q4.** Thinking about the past week, on a TYPICAL DAY, how much time did your baby spend watching television programs, videos/internet clips or movies on a television, computer or portable/mobile device such as iPad, tablet or smartphone?

- ◯ 0 min per day [0]
- ◯ Between 1 and 15 min per day [1]
- ◯ Between 15 and 30 min per day [2]
- ◯ Between 30 and 60 min per day [3]
- ◯ Between 1 and 1½ hrs per day [4]
- ◯ Between 1½ and 2 hrs per day [5]
- ◯ More than 2 hrs per day [6]

**Q5** Thinking about the past week, on a TYPICAL DAY, how much time did your baby spend playing games, looking at photos, or video chatting (e.g. FaceTime, Zoom, Skype) on a screen-based device such as a computer or laptop, video game console, iPad, tablet, or smartphone?

- ◯ 0 min per day [0]
- ◯ Between 1 and 15 min per day [1]
- ◯ Between 15 and 30 min per day [2]
- ◯ Between 30 and 60 min per day [3]
- ◯ Between 1 and 1½ hrs per day [4]
- ◯ Between 1½ and 2 hrs per day [5]
- ◯ More than 2 hrs per day [6]

## This section is about your baby's sleep

**Q6.** Thinking about the past week, on a TYPICAL NIGHT, how much time did your baby sleep in total during the night?

- ◯ Less than 6 hrs per night [1]
- ◯ Between 6 and 8 hrs per night [2]
- ◯ Between 8 and 10 hrs per night [3]
- ◯ Between 10 and 12 hrs per night [4]
- ◯ Between 12 and 14 hrs per night [5]
- ◯ More than 14 hrs per night [6]

**Q7.** Thinking about the past week, on a TYPICAL DAY, how much time did your baby sleep in total during the day?

- ◯ Less than 1 hr per day [1]
- ◯ Between 1 and 2 hrs per day [2]
- ◯ Between 2 and 3 hrs per day [3]
- ◯ Between 3 and 4 hrs per day [4]
- ◯ More than 4 hrs per day [5]

--- Thank you for completing the MBQ ---

# Calculation of outcome variables

| Outcome variable | Calculation |
|---|---|
| **TUMMY TIME** | |
| Usual tummy time (minutes/bout) | Q1B: [1] = 2.5; [2] = 7.5; [3] = 12.5; [4] = 18; [5] = 23; [6] = 28; [7] = 30 |
| Total tummy time (minutes/day) | ______times (Q1A) x usual tummy time (Q1B) |
| **ACTIVE PLAY** | |
| Total Active Play (minutes/day) | Q2: [0] = 0; [1] = 7.5; [2] = 22.5; [3] = 45; [4] = 75; [5] = 105; [6] = 120 |
| **RESTRAINED TIME** | |
| Usual restrained time (minutes/bout) | Q3B: [1] = 7.5; [2] = 22.5; [3] = 37.5; [4] = 52.5; [5] = 75; [6] = 105; [7] = 120 |
| Total restrained time (minutes/day) | ______times (Q3A) x usual restrained time (Q3B) |
| **SCREEN TIME** | |
| Non-interactive screen time (minutes/day) | Q4: [0] = 0; [1] = 7.5; [2] = 22.5; [3] = 45; [4] = 75; [5] = 105; [6] = 120 |
| Interactive screen time (minutes/day) | Q5: [0] = 0; [1] = 7.5; [2] = 22.5; [3] = 45; [4] = 75; [5] = 105; [6] = 120 |
| Total screen time (minutes/day) | Non-interactive screen time + Interactive screen time |
| **SLEEP** | |
| Night sleep (minutes) | Q6: [1] = 300; [2] = 420; [3] = 540; [4] = 660; [5] = 780; [6] = 840 |
| Day sleep (minutes) | Q7: [1] = 30; [2] = 90; [3] = 150; [4] = 210; [5] = 240 |
| Total sleep (minutes per 24 hours) | Night Sleep + Day Sleep |

# Truncation of extreme values

Implausible or extreme values for the following outcome variables are truncated (that is recoded) to the value equivalent of the 95th percentile from the validation study dataset.

| Outcome variable | Truncated value |
|---|---|
| Total tummy time | 180 minutes per day |
| Total restrained time | 360 minutes per day |

# Adherence to Guidelines

### Physical Activity

Tummy time (for babies who are yet to roll) – at least 30 minutes of tummy time per day.

Active Play (for babies who can roll) – at least 30 minutes of active play per day.

### Restrained Time

Not more than 60 minutes (1 hour) at a time of restrained time (such as in a stroller, car seat or highchair).

### Screen time

No screen time for babies/children under 2 years.

### Sleep

Age = 0 – 3 months: At least 840 minutes (14 hours) per day.

Age = 4 – 11 months: At least 720 minutes (12 hours) per day.

Age = 12 months or older: At least 660 minutes (11 hours) per day.

### 24-hour movement guidelines

**For babies who are yet to roll:** meets all 3 guidelines for tummy time, screen time and sleep.

**For babies who can roll** meets all 3 guidelines for active play, screen time and sleep.

# MBQ Index

| MBQ Index variable | Calculation |
|---|---|
| **TUMMY TIME AND ACTIVE PLAY** | |
| Tummy time index | IF ‘Total tummy time’ > 40,<br>Tummy time index = 100<br>IF ‘Total tummy time’ <= 40,<br>Tummy time index =<br>(‘Total tummy time’ /40) x 100 |
| Active Play index | IF ‘Total Active Play’ > 40,<br>Active Play index = 100<br>IF ‘Total Active Play’ <= 40,<br>Active Play index =<br>(‘Total Active Play’ /40) x 100 |
| **RESTRAINED TIME** | |
| Restrained time index | IF Q3B = 1, Restrained time index = 100<br>IF Q3B = 2, Restrained time index = 95<br>IF Q3B = 3, Restrained time index = 85<br>IF Q3B = 4, Restrained time index = 75<br>IF Q3B = 5, Restrained time index = 50<br>IF Q3B = 6, Restrained time index = 25<br>IF Q3B = 7, Restrained time index = 0 |
| **SCREEN TIME** | |
| Non-interactive screen time index | IF Q4 = 0, Non-interactive screen time index = 100<br>IF Q4 = 1, Non-interactive screen time index = 75<br>IF Q4 = 2, Non-interactive screen time index = 50<br>IF Q4 = 3, Non-interactive screen time index = 25<br>IF Q4 = 4, Non-interactive screen time index = 10<br>IF Q4 = 5, Non-interactive screen time index = 5<br>IF Q4 = 6, Non-interactive screen time index = 0 |
| Interactive screen time index | IF Q5 = 0, Interactive screen time index = 100<br>IF Q5 = 1, Interactive screen time index = 75<br>IF Q5 = 2, Interactive screen time index = 50<br>IF Q5 = 3, Interactive screen time index = 25<br>IF Q5 = 4, Interactive screen time index = 10<br>IF Q5 = 5, Interactive screen time index = 5<br>IF Q5 = 6, Interactive screen time index = 0 |
| **SLEEP** | |
| Sleep index raw score | **IF baby is aged 0 to 3 months [1].** |

| | |
|---|---|
| | Sleep index raw score =<br>((‘Total sleep’ – 300)/ 720) *100<br>(Index = 100 when Total Sleep is 17 hours or more)<br><br>**IF baby is aged 4 to 11 months [2].**<br>Sleep index raw score =<br>((‘Total sleep’ – 300)/ 560) *100<br>(Index = 100 when Total Sleep is 14.3 hours or more)<br>**IF baby is aged 12 months or older [3].**<br>Sleep index raw score =<br>((‘Total sleep’ – 300)/ 480) *100<br>(Index = 100 when Sleep is 13 hours or more) |
| Sleep index | IF Sleep index raw score > 100, Sleep index = 100<br>IF Sleep index raw score <= 100, Sleep index = Sleep index raw score |
| **MBQ Index – Summary Score** | |
| MBQ-B index summary score | MBQ Index summary score is based on the average of index scores for each behaviour.<br>**IF baby is yet to roll [0]:**<br>MBQ-B index summary score = Mean (Tummy time index, Restrained time index, Non-interactive screen time index, Interactive screen time index, Sleep index)<br>**IF baby can roll [1]:**<br>MBQ-B index summary score = Mean (Active play index, Restrained time index, Non-interactive screen time index, Interactive screen time index, Sleep index) |

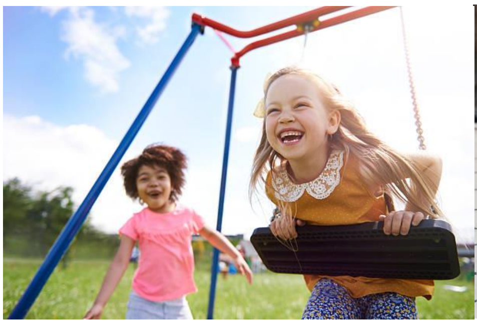
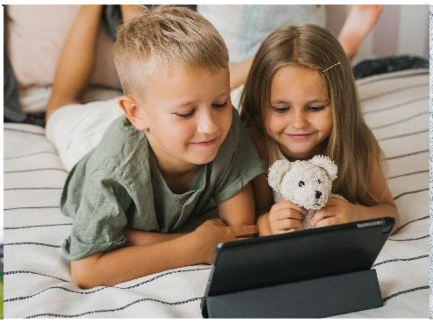
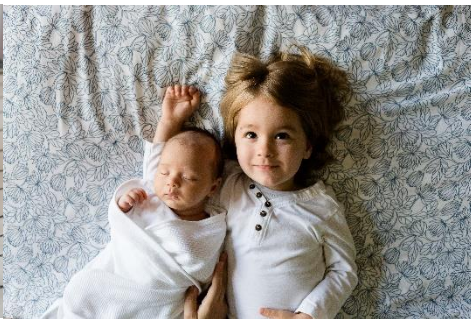

# Movement Behaviour Questionnaire

# Child Open Version

# (MBQ-C)

## OVERVIEW

- The MBQ is a validated rapid assessment tool for measuring movement behaviours in children aged 0 – 5 years.
- The MBQ-C measures active play, screen time and sleep.

## TARGET POPULATION

- For children aged up to 5 years *who can walk*.

## HOW IT IS MEASURED

- 9 items
- Open-ended questions
- Self-report
- Hard-copy or electronic completion (REDCap data dictionary available upon request)
- Supplemented by the User guide for administration and scoring (hard copy or REDCap versions available)

Stewart. G. Trost

Contact for information: s.trost@uq.edu.au

Date: ____ / ____ / ______

# Movement Behaviour Questionnaire (MBQ-C)

## DIRECTIONS

This survey will ask you questions about your child's movement behaviours (activity, screen time, and sleep) on a typical day.

A typical day is a day when your child does things they normally do.

For questions about how much time your child spends in these behaviours, please provide an answer to both hours and minutes, e.g., 2 hours 0 minutes, 0 hours 30 minutes.

Please respond to all the questions as best as you can.

---

How old is your child?

- ◯ 1 year old [1]
- ◯ 2 years old [2]
- ◯ 3 years old [3]
- ◯ 4 years old [4]
- ◯ 5 years old [5]

This section is about your child's physical activity

**Q1A.** Thinking about the past week, on a TYPICAL **WEEKDAY**, how much time did your child spend in active play? *Active play includes activities such as walking, running, dancing, climbing, playing with balls, riding bikes or scooters, or swimming.*

☐ hours ☐ minutes **If you answered 0 hours/min → Skip to question 2A**

**Q1B.** Of this time, how much was spent doing vigorous activities such as running, jumping, dancing, riding bikes or scooters? Please note, the time provided must be less than your previous answer.

☐ hours ☐ minutes

---

**Q2A.** Thinking about the past week, on a TYPICAL **WEEKEND DAY**, how much time did your child spend in active play? *Active play includes activities such as walking, running, dancing, climbing, playing with balls, riding bikes or scooters, or swimming.*

☐ hours ☐ minutes **If you answered 0 hours/min → Skip to question 3A**

**Q2B.** Of this time, how much was spent doing vigorous activities such as running, jumping, dancing, riding bikes or scooters? Please note, the time provided must be less than your previous answer.

☐ hours ☐ minutes

---

## This section is about your child's screen time

**Q3A.** Thinking about the past week, on a TYPICAL **WEEKDAY**, how much time did your child spend watching television programs, videos/internet clips or movies on a television, computer, or portable/mobile device such as iPad, tablet or smartphone?

[ ] hours [ ] minutes **If you answered 0 hours/min → Skip to question 4A**

**Q3B.** Of this time, how much time did they spend watching television programs, videos/internet clips, or movies while standing? Please note, the time provided must be less than your previous answer.

[ ] hours [ ] minutes

---

**Q4A.** Thinking about the past week, on a TYPICAL **WEEKEND DAY**, how much time did your child spend watching television programs, videos/internet clips or movies on a television, computer, or portable/mobile device such as iPad, tablet or smartphone?

[ ] hours [ ] minutes **If you answered 0 hours/min → Skip to question 5A**

**Q4B.** Of this time, how much time did they spend watching television programs, videos/internet clips, or movies while standing? Please note, the time provided must be less than your previous answer.

[ ] hours [ ] minutes

---

**Q5A.** Thinking about the past week, on a TYPICAL **WEEKDAY**, how much time did your child spend playing games, looking at photos, or video chatting (e.g. FaceTime, Zoom, Skype) on a screen-based device such as a computer or laptop, video game console, iPad, tablet, or smartphone?

[ ] hours [ ] minutes **If you answered 0 hours/min → Skip to question 6A**

**Q5B.** Of this time, how much time did they spend playing games, looking at photos, or video chatting (e.g. FaceTime, Zoom, Skype) while standing? Please note, the time provided must be less than your previous answer.

[ ] hours [ ] minutes

---

**Q6A.** Thinking about the past week, on a TYPICAL **WEEKEND DAY**, how much time did your child spend playing games, looking at photos, or video chatting (e.g. FaceTime, Zoom, Skype) on a screen-based device such as a computer or laptop, video game console, iPad, tablet, or smartphone?

[ ] hours [ ] minutes **If you answered 0 hours/min → Skip to question 7**

**Q6B.** Of this time, how much time did they spend playing games, looking at photos, or video chatting (e.g. FaceTime, Zoom, Skype) while standing? Please note, the time provided must be less than your previous answer.

[ ] hours [ ] minutes

This section is about your child's sleep

**Q7.** Thinking about the past week, on a TYPICAL NIGHT, how much time did your child sleep in total during the night?

[ ] hours [ ] minutes

**Q8.** Thinking about the past week, on a TYPICAL DAY, how much time did your child sleep in total during the day?

[ ] hours [ ] minutes

**Q9.** In a TYPICAL WEEK, how often does your child have a regular bedtime routine (e.g., bath, story)?

[ ] times

--- Thank you for completing the MBQ ---

# Calculation of outcome variables

| Outcome variable | Calculation |
|---|---|
| **PHYSICAL ACTIVITY** | |
| Weekday total active play (minutes/day) | Q1A (______hours x 60) + ______minutes |
| Weekday energetic play (minutes/day) | Q1B (______hours x 60) + ______minutes |
| Weekend day total active play (minutes/day) | Q2A (______hours x 60) + ______minutes |
| Weekend day energetic play (minutes/day) | Q2B (______hours x 60) + ______minutes |
| Weighted average of total active play (minutes/day) | ((Weekday total active play x 5) + (Weekend day total active play x 2)) / 7 |
| Weighted average of energetic play (minutes/day) | ((Weekday energetic play x 5) + (Weekend day energetic play x 2)) / 7 |
| **SCREEN TIME** | |
| Weekday passive screen time (minutes/day) | Q3A (______hours x 60) + ______minutes |
| Weekday passive screen time while standing (minutes/day) | Q3B (______hours x 60) + ______minutes |
| Weekend day passive screen time (minutes/day) | Q4A (______hours x 60) + ______minutes |
| Weekend day passive screen time while standing (minutes/day) | Q4B (______hours x 60) + ______minutes |
| Weekday SEDENTARY passive screen time (minutes/day) | Weekday passive screen time - Weekday passive screen time while standing<br>NOTE. If weekday passive screen time while standing exceeds total passive screen time, 'Weekday passive screen time' should be used to calculate the weighted average for Weekday SEDENTARY passive screen time. |

| | |
|---|---|
| Weekend day SEDENTARY passive screen time (minutes/day) | Weekend day passive screen time - Weekend day passive screen time while standing<br>NOTE. If weekend day passive screen time while standing exceeds total passive screen time, ‘Weekend day passive screen time’ should be used to calculate the weighted average for Weekend day SEDENTARY passive screen time. |
| Weighted average of passive screen time (minutes/day) | ((Weekday passive screen time x 5) + (Weekend day passive screen time x 2)) / 7 |
| Weighted average of SEDENTARY passive screen time (minutes/day) | ((Weekday sedentary passive screen time x 5) + (Weekend day sedentary passive screen time x 2)) / 7 |
| Weekday interactive screen time (minutes/day) | Q5A (______hours x 60) + ______minutes |
| Weekday interactive screen time while standing (minutes/day) | Q5B (______hours x 60) + ______minutes |
| Weekend day interactive screen time (minutes/day) | Q6A (______hours x 60) + ______minutes |
| Weekend day interactive screen time while standing (minutes/day) | Q6B (______hours x 60) + ______minutes |
| Weekday SEDENTARY interactive screen time (minutes/day) | Weekday interactive screen time - Weekday interactive screen time while standing<br>NOTE. If weekday interactive screen time while standing exceeds total interactive screen time, ‘Weekday interactive screen time’ should be used to calculate the weighted average for Weekday SEDENTARY interactive screen time. |
| Weekend day SEDENTARY interactive screen time (minutes/day) | Weekend day interactive screen time - Weekend day interactive screen time while standing<br>NOTE. If weekend day interactive screen time while standing exceeds total interactive screen time, ‘Weekend day interactive screen time’ should be used to calculate the weighted average for Weekend day SEDENTARY interactive screen time. |
| Weighted average of interactive screen time | ((Weekday interactive screen time x 5) + (Weekend day interactive screen time x 2)) / 7 |

| | |
|---|---|
| Weighted average of SEDENTARY interactive screen time | ((Weekday SEDENTARY interactive screen time x 5) + (Weekend day SEDENTARY interactive screen time x 2)) / 7 |
| Weighted average of total screen time | Weighted average of passive screen time + Weighted average of interactive screen time |
| Weighted average of total SEDENTARY screen time | Weighted average of SEDENTARY passive screen time + Weighted average of SEDENTARY interactive screen time |
| **SLEEP** | |
| Night sleep (minutes) | Q7 (______hours x 60) + ______minutes |
| Day sleep (minutes) | Q8 (______hours x 60) + ______minutes |
| Total sleep (minutes per 24 hours) | Night Sleep + Day Sleep |

## Truncation of extreme values

Implausible or extreme values for the following outcome variables are truncated (that is recoded) to the value equivalent of the 95th percentile from the validation study dataset.

| **Outcome variable** | **Truncated value** |
|---|---|
| Weekday total active play | 480 minutes per day |
| Weekend day total active play | 480 minutes per day |
| Weekday energetic play | 360 minutes per day |
| Weekend day energetic play | 360 minutes per day |

# Adherence to Guidelines

## Physical Activity

At least 3 hours of total physical activity per day, including 1 hour of energetic play.

## Screen time

Age = under 2 years: No screen time for babies/children.

Age = 2 – 5 years: No more than 1 hour per day of sedentary screen time.

## Sleep

Age = under 3 years: At least 660 minutes (11 hours) per day.

Age = 3 years or older: At least 600 minutes (10 hours) per day.

## 24-hour movement guidelines

Meets all 3 guidelines for physical activity, screen time and sleep.

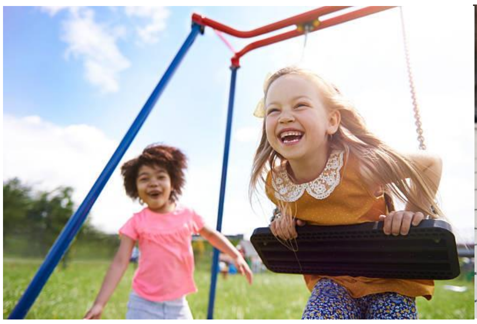
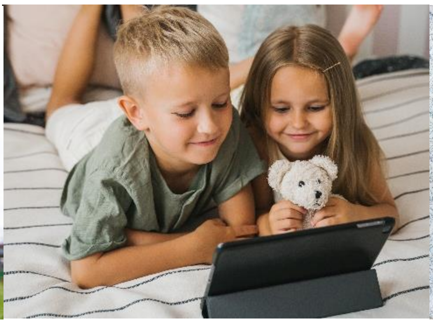
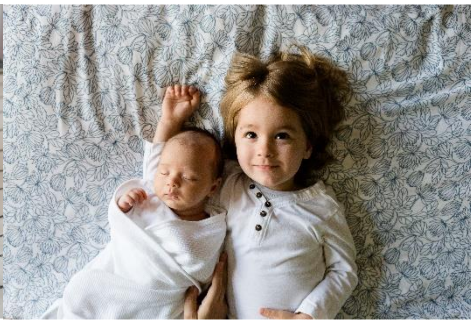

# Movement Behaviour Questionnaire

# Child Closed Version

# (MBQ-C)

## OVERVIEW

- The MBQ is a validated rapid assessment tool for measuring movement behaviours in children aged 0 – 5 years.
- The MBQ-C measures active play, screen time and sleep.

## TARGET POPULATION

- For children aged up to 5 years *who can walk*.

## HOW IT IS MEASURED

- 9 items
- Closed questions
- Self-report
- Hard-copy or electronic completion (REDCap data dictionary available upon request)
- Supplemented by the User guide for administration and scoring (hard copy or REDCap versions available)

Stewart. G. Trost

Contact for information: s.trost@uq.edu.au

Date: ____ / ____ / ______

# Movement Behaviour Questionnaire (MBQ-C)

## DIRECTIONS

This survey will ask you questions about your child's movement behaviours (activity, screen time, and sleep) on a typical day.

A typical day is a day when your child does things they normally do.

Please respond to all the questions as best as you can.

---

How old is your child?

- ◯ 1 year old [1]
- ◯ 2 years old [2]
- ◯ 3 years old [3]
- ◯ 4 years old [4]
- ◯ 5 years old [5]

## This section is about your child's physical activity

**Q1A.** Thinking about the past week, on a TYPICAL **WEEKDAY**, how much time did your child spend in active play? *Active play includes activities such as walking, running, dancing, climbing, playing with balls, riding bikes or scooters, or swimming.*

- ◯ 0 min per day [0] **→ Skip to question 2A**
- ◯ Between 1 and 30 min per day [1]
- ◯ Between 30 and 60 min per day [2]
- ◯ Between 1 and 2 hrs per day [3]
- ◯ Between 2 and 3 hrs per day [4]
- ◯ Between 3 and 4 hrs per day [5]
- ◯ More than 4 hrs per day [6]

**Q1B.** Of this time, how much was spent doing vigorous activities such as running, jumping, dancing, riding bikes or scooters? Please note, the time provided must be less than your previous answer.

- ◯ 0 min per day [0]
- ◯ Between 1 and 15 min per day [1]
- ◯ Between 15 and 30 min per day [2]
- ◯ Between 30 and 60 min per day [3]
- ◯ Between 1 and 1½ hrs per day [4]
- ◯ Between 1½ and 2 hrs per day [5]
- ◯ More than 2 hrs per day [6]

---

**Q2A.** Thinking about the past week, on a TYPICAL **WEEKEND DAY**, how much time did your child spend in active play? *Active play includes activities such as walking, running, dancing, climbing, playing with balls, riding bikes or scooters, or swimming.*

- ◯ 0 min per day [0] **→ Skip to question 3A**
- ◯ Between 1 and 30 min per day [1]
- ◯ Between 30 and 60 min per day [2]
- ◯ Between 1 and 2 hrs per day [3]
- ◯ Between 2 and 3 hrs per day [4]
- ◯ Between 3 and 4 hrs per day [5]
- ◯ More than 4 hrs per day [6]

**Q2B.** Of this time, how much was spent doing vigorous activities such as running, jumping, dancing, riding bikes or scooters? Please note, the time provided must be less than your previous answer.

- ◯ 0 min per day [0]
- ◯ Between 1 and 15 min per day [1]
- ◯ Between 15 and 30 min per day [2]
- ◯ Between 30 and 60 min per day [3]
- ◯ Between 1 and 1½ hrs per day [4]
- ◯ Between 1½ and 2 hrs per day [5]
- ◯ More than 2 hrs per day [6]

## This section is about your child's screen time

**Q3A.** Thinking about the past week, on a TYPICAL **WEEKDAY**, how much time did your child spend watching television programs, videos/internet clips or movies on a television, computer, or portable/mobile device such as iPad, tablet or smartphone?

- ◯ 0 min per day [0] **→ Skip to question 4A**
- ◯ Between 1 and 15 min per day [1]
- ◯ Between 15 and 30 min per day [2]
- ◯ Between 30 and 60 min per day [3]
- ◯ Between 1 and 1½ hrs per day [4]
- ◯ Between 1½ and 2 hrs per day [5]
- ◯ Between 2 and 3 hrs per day [6]
- ◯ More than 3 hrs per day [7]

**Q3B.** Of this time, how much time did they spend watching television programs, videos/internet clips, or movies while standing? Please note, the time provided must be less than your previous answer.

- ◯ 0 min per day [0]
- ◯ Between 1 and 15 min per day [1]
- ◯ Between 15 and 30 min per day [2]
- ◯ Between 30 and 60 min per day [3]
- ◯ Between 1 and 1½ hrs per day [4]
- ◯ Between 1½ and 2 hrs per day [5]
- ◯ Between 2 and 3 hrs per day [6]
- ◯ More than 3 hrs per day [7]

**Q4A.** Thinking about the past week, on a TYPICAL **WEEKEND DAY**, how much time did your child spend watching television programs, videos/internet clips or movies on a television, computer, or portable/mobile device such as iPad, tablet or smartphone?

◯ 0 min per day [0] **→ Skip to question 5A**
◯ Between 1 and 15 min per day [1]
◯ Between 15 and 30 min per day [2]
◯ Between 30 and 60 min per day [3]
◯ Between 1 and 1½ hrs per day [4]
◯ Between 1½ and 2 hrs per day [5]
◯ Between 2 and 3 hrs per day [6]
◯ More than 3 hrs per day [7]

**Q4B.** Of this time, how much time did they spend watching television programs, videos/internet clips, or movies while standing? Please note, the time provided must be less than your previous answer.

◯ 0 min per day [0]
◯ Between 1 and 15 min per day [1]
◯ Between 15 and 30 min per day [2]
◯ Between 30 and 60 min per day [3]
◯ Between 1 and 1½ hrs per day [4]
◯ Between 1½ and 2 hrs per day [5]
◯ Between 2 and 3 hrs per day [6]
◯ More than 3 hrs per day [7]

---

**Q5A.** Thinking about the past week, on a TYPICAL **WEEKDAY**, how much time did your child spend playing games, looking at photos, or video chatting (e.g. FaceTime, Zoom, Skype) on a screen-based device such as a computer or laptop, video game console, iPad, tablet, or smartphone?

◯ 0 min per day [0] **→ Skip to question 6A**
◯ Between 1 and 15 min per day [1]
◯ Between 15 and 30 min per day [2]
◯ Between 30 and 60 min per day [3]
◯ Between 1 and 1½ hrs per day [4]
◯ Between 1½ and 2 hrs per day [5]
◯ Between 2 and 3 hrs per day [6]
◯ More than 3 hrs per day [7]

**Q5B.** Of this time, how much time did they spend playing games, looking at photos, or video chatting (e.g. FaceTime, Zoom, Skype) while standing? Please note, the time provided must be less than your previous answer.

◯ 0 min per day [0]
◯ Between 1 and 15 min per day [1]
◯ Between 15 and 30 min per day [2]
◯ Between 30 and 60 min per day [3]
◯ Between 1 and 1½ hrs per day [4]
◯ Between 1½ and 2 hrs per day [5]
◯ Between 2 and 3 hrs per day [6]
◯ More than 3 hrs per day [7]

---

**Q6A.** Thinking about the past week, on a TYPICAL **WEEKEND DAY**, how much time did your child spend playing games, looking at photos, or video chatting (e.g. FaceTime, Zoom, Skype) on a screen-based device such as a computer or laptop, video game console, iPad, tablet, or smartphone?

- ◯ 0 min per day [0] **→ Skip to question 7A**
- ◯ Between 1 and 15 min per day [1]
- ◯ Between 15 and 30 min per day [2]
- ◯ Between 30 and 60 min per day [3]
- ◯ Between 1 and 1½ hrs per day [4]
- ◯ Between 1½ and 2 hrs per day [5]
- ◯ Between 2 and 3 hrs per day [6]
- ◯ More than 3 hrs per day [7]

**Q6B.** Of this time, how much time did they spend playing games, looking at photos, or video chatting (e.g. FaceTime, Zoom, Skype) while standing? Please note, the time provided must be less than your previous answer.

- ◯ 0 min per day [0]
- ◯ Between 1 and 15 min per day [1]
- ◯ Between 15 and 30 min per day [2]
- ◯ Between 30 and 60 min per day [3]
- ◯ Between 1 and 1½ hrs per day [4]
- ◯ Between 1½ and 2 hrs per day [5]
- ◯ Between 2 and 3 hrs per day [6]
- ◯ More than 3 hrs per day [7]

## This section is about your child's sleep

**Q7.** Thinking about the past week, on a TYPICAL NIGHT, how much time did your child sleep in total during the night?

- ◯ Less than 6 hrs per night [1]
- ◯ Between 6 and 8 hrs per night [2]
- ◯ Between 8 and 10 hrs per night [3]
- ◯ Between 10 and 12 hrs per night [4]
- ◯ Between 12 and 14 hrs per night [5]
- ◯ More than 14 hrs per night [6]

**Q8.** Thinking about the past week, on a TYPICAL DAY, how much time did your child sleep in total during the day?

- ◯ 0 hrs per day [0]
- ◯ Less than 1 hr per day [1]
- ◯ Between 1 and 2 hrs per day [2]
- ◯ Between 2 and 3 hrs per day [3]
- ◯ Between 3 and 4 hrs per day [4]
- ◯ More than 4 hrs per day [5]

**Q9.** In a TYPICAL WEEK, how often does your child have a regular bedtime routine (e.g., bath, story)?

- ◯ Never [0]
- ◯ 1 – 2 nights per week [1]
- ◯ 3 – 4 nights per week [2]
- ◯ 5 – 6 nights per week [3]
- ◯ Every night [4]

--- Thank you for completing the MBQ ---

# Calculation of outcome variables

| Outcome variable | Calculation |
|---|---|
| **PHYSICAL ACTIVITY** | |
| Weekday total active play (minutes/day) | Q1A: [0] = 0; [1] = 15; [2] = 45; [3] = 90; [4] = 150; [5] = 210; [6] = 240 |
| Weekday energetic play (minutes/day) | Q1B: [0] = 0; [1] = 7.5; [2] = 22.5; [3] = 45; [4] = 75; [5] = 105; [6] = 120 |
| Weekend day total active play (minutes/day) | Q2A: [0] = 0; [1] = 15; [2] = 45; [3] = 90; [4] = 150; [5] = 210; [6] = 240 |
| Weekend day energetic play (minutes/day) | Q2B: [0] = 0; [1] = 7.5; [2] = 22.5; [3] = 45; [4] = 75; [5] = 105; [6] = 120 |
| Weighted average of energetic play (minutes/day) | ((Weekday energetic play x 5) + (Weekend day energetic play x 2)) / 7 |
| Weighted average of total active play (minutes/day) | ((Weekday total active play x 5) + (Weekend day total active play x 2)) / 7 |
| **SCREEN TIME** | |
| Weekday passive screen time (minutes/ weekday) | Q3A: [0] = 0; [1] = 7.5; [2] = 22.5; [3] = 45; [4] = 75; [5] = 105; [6] = 150; [7] = 240 |
| Weekday passive screen time while standing (minutes/ weekday) | Q3B: [0] = 0; [1] = 7.5; [2] = 22.5; [3] = 45; [4] = 75; [5] = 105; [6] = 150; [7] = 240 |
| Weekend day passive screen time (minutes/ weekend day) | Q4A: [0] = 0; [1] = 7.5; [2] = 22.5; [3] = 45; [4] = 75; [5] = 105; [6] = 150; [7] = 240 |
| Weekend day passive screen time while standing (minutes/ weekend day) | Q4B: [0] = 0; [1] = 7.5; [2] = 22.5; [3] = 45; [4] = 75; [5] = 105; [6] = 150; [7] = 240 |

| | |
|---|---|
| Weekday SEDENTARY passive screen time (minutes/day) | Weekday passive screen time - Weekday passive screen time while standing<br><br>NOTE. If weekday passive screen time while standing exceeds total passive screen time, ‘Weekday passive screen time’ should be used to calculate the weighted average for Weekday SEDENTARY passive screen time. |
| Weekend day SEDENTARY passive screen time (minutes/day) | Weekend day passive screen time - Weekend day passive screen time while standing<br><br>NOTE. If weekend day passive screen time while standing exceeds total passive screen time, ‘Weekend day passive screen time’ should be used to calculate the weighted average for Weekend day SEDENTARY passive screen time. |
| Weekday interactive screen time (minutes/ weekday) | Q5A: [0] = 0; [1] = 7.5; [2] = 22.5; [3] = 45; [4] = 75; [5] = 105; [6] = 150; [7] = 240 |
| Weekday interactive screen time while standing (minutes/ weekday) | Q5B: [0] = 0; [1] = 7.5; [2] = 22.5; [3] = 45; [4] = 75; [5] = 105; [6] = 150; [7] = 240 |
| Weekend day interactive screen time (minutes/ weekend day) | Q6A: [0] = 0; [1] = 7.5; [2] = 22.5; [3] = 45; [4] = 75; [5] = 105; [6] = 150; [7] = 240 |
| Weekend day interactive screen time while standing (minutes/ weekend day) | Q6B: [0] = 0; [1] = 7.5; [2] = 22.5; [3] = 45; [4] = 75; [5] = 105; [6] = 150; [7] = 240 |
| Weekday SEDENTARY interactive screen time (minutes/day) | Weekday interactive screen time - Weekday interactive screen time while standing<br><br>NOTE. If weekday interactive screen time while standing exceeds total interactive screen time, ‘Weekday interactive screen time’ should be used to calculate the weighted average for Weekday SEDENTARY interactive screen time. |
| Weekend day SEDENTARY interactive screen time (minutes/day) | Weekend day interactive screen time - Weekend day interactive screen time while standing<br><br>NOTE. If weekend day interactive screen time while standing exceeds total interactive screen time, ‘Weekend day interactive screen time’ should be used to calculate the weighted average for Weekend day SEDENTARY interactive screen time. |

| | |
|---|---|
| Weighted average of passive screen time (minutes/day) | ((Weekday passive screen time x 5) + (Weekend day passive screen time x 2)) / 7 |
| Weighted average of SEDENTARY passive screen time (minutes/day) | ((Weekday SEDENTARY passive screen time x 5) + (Weekend day SEDENTARY passive screen time x 2)) / 7 |
| Weighted average of interactive screen time (minutes/day) | ((Weekday interactive screen time x 5) + (Weekend day interactive screen time x 2)) / 7 |
| Weighted average of SEDENTARY interactive screen time (minutes/day) | ((Weekday SEDENTARY interactive screen time x 5) + (Weekend day SEDENTARY interactive screen time x 2)) / 7 |
| Weighted average of total screen time (minutes/day) | Weighted average of passive screen time + Weighted average of interactive screen time |
| Weighted average of total SEDENTARY screen time (minutes/day) | Weighted average of SEDENTARY passive screen time + Weighted average of SEDENTARY interactive screen time |
| **SLEEP** | |
| Night sleep (minutes) | Q7: [1] = 360; [2] = 420; [3] = 540; [4] = 660; [5] = 780; [6] = 840 |
| Day sleep (minutes) | Q8: [0] = 0; [1] = 30; [2] = 90; [3] = 150; [4] = 210; [5] = 240 |
| Total sleep (minutes per 24 hours) | Night Sleep + Day Sleep |

# Truncation of extreme values

Implausible or extreme values for the following outcome variables are truncated (that is recoded) to the value equivalent of the 95th percentile from the validation study dataset.

| **Outcome variable** | **Truncated value** |
|---|---|
| Weekday total active play | 480 minutes per day |
| Weekend day total active play | 480 minutes per day |
| Weekday energetic play | 360 minutes per day |
| Weekend day energetic play | 360 minutes per day |

# Adherence to Guidelines

## Physical Activity

At least 3 hours of total physical activity per day, including 1 hour of energetic play.

## Screen time

Age = under 2 years: No screen time for babies/children.

Age = 2 – 5 years: No more than 1 hour per day of sedentary screen time.

## Sleep

Age = under 3 years: At least 660 minutes (11 hours) per day.

Age = 3 years or older: At least 600 minutes (10 hours) per day.

## 24-hour movement guidelines

Meets all 3 guidelines for physical activity, screen time and sleep.

# MBQ Index

| MBQ Index variable | Calculation |
|---|---|
| **PHYSICAL ACTIVITY** | |
| Physical activity index | = (‘Weighted average of total active play’/ 240) x 100 |
| Energetic play index | IF ‘Weighted average of energetic play’ > 80,<br>Energetic play index = 100<br>IF ‘Weighted average of energetic play’ <= 80,<br>Energetic play index =<br>(‘Weighted average of energetic play’ /80) x 100 |
| **SCREEN TIME** | |
| Passive screen time index | Passive screen time index =<br>(1 - (‘Weighted average of passive screen time’/ 240)) x 100 |
| SEDENTARY passive screen time index | SEDENTARY passive screen time index =<br>(1 - (‘Weighted average of SEDENTARY passive screen time’/ 240)) x 100 |
| Interactive screen time index | Interactive screen time index =<br>(1 - (‘Weighted average of interactive screen time’/ 240)) x 100 |
| SEDENTARY interactive screen time index | SEDENTARY interactive screen time index =<br>(1 - (‘Weighted average of SEDENTARY interactive screen time’/ 240)) x 100 |
| **SLEEP** | |
| Sleep index raw score | **IF child is younger than 3 years [1] or [2].**<br>Sleep index raw score =<br>((‘Total sleep’ - 300)/ 480) x 100<br>(Index = 100 when Total Sleep is 13 hours or more; 75 when Total Sleep is 11 or more)<br>**IF child is 3 years or older [3] or [4] or [5].**<br>Sleep index raw score =<br>((‘Total sleep’ - 240)/ 480) x 100<br>(Index = 100 when Total Sleep is 12 hours or more; 75 when Total Sleep is 10 hours or more) |
| Sleep index | IF Sleep index raw score > 100, Sleep index = 100<br>IF Sleep index raw score <= 100, Sleep index = Sleep index raw score |

| | |
|---|---|
| Sleep routine index | Sleep routine index = (Q9/ 4) x 100 |
| **MBQ Index – Summary Score** | |
| MBQ-C index summary score | MBQ Index summary score is based on the average of index scores for each behaviour.<br>MBQ-C index summary score = mean (Physical activity index, Energetic play index, Passive screen time index, SEDENTARY passive screen time index, Interactive screen time index, SEDENTARY interactive screen time index, Sleep index, Sleep routine index) |
