# Supplementary material for: Reliability and validity of rapid assessment tools for measuring 24-hour movement behaviours in children aged 0–5 years: the Movement Behaviour Questionnaire Baby (MBQ-B) and child (MBQ-C)
Source: Int J Behav Nutr Phys Act. 2024 Apr 23;21:43. doi: 10.1186/s12966-024-01596-5 (PMC11041005; doi:10.1186/s12966-024-01596-5)
Supplement: Supplementary file 3 — Supplementary Material 3 [file 12966_2024_1596_MOESM3_ESM.pdf]

24-hour Activity Log for Babies

DIRECTIONS: Use this form to record your baby's sleep, activity and screen time over a 24-hour period. This log may be easier to fill out as you go.

An example is shown below:

Please **circle** which day this log has been completed on: MONDAY TUESDAY (WEDNESDAY) THURSDAY FRIDAY SATURDAY SUNDAY

Please record the **date** this log has been completed on: _22_ / _04_ / 2021 Is this a childcare day? ☐ Yes ☑ No

	MORNING																															
**ACTIVITY**	**5 AM**				**6 AM**				**7 AM**				**8 AM**				**9 AM**				**10 AM**				**11 AM**				**12 PM**			
**What is your baby doing?**	00	15	30	45	00	15	30	45	00	15	30	45	00	15	30	45	00	15	30	45	00	15	30	45	00	15	30	45	00	15	30	45
1. Sleeping	→	→	→	→	→	→	→	→													→	→	→	→	→	→	→	→				
2. Tummy time												→																				
3. Supervised active play													→	→																		
4. Restrained time										→	→																			→	→	
5. Watching TV, videos/internet clips or movies															→	→																
6. Playing games, looking at photos, or video chatting on screen-based devices																																

DETAILED INSTRUCTIONS: In the appropriate column, draw an arrow through each 15-minute time period your baby's **main activity** was:

1. **Sleeping**
2. **Tummy time** – times when your baby is awake and placed on their tummy for playtime while you are watching them
3. **Supervised active play** – could be crawling on the floor with your baby, rolling around the floor with your baby, playing at the park, dancing with your baby, chasing your baby
4. **Restrained time** – time spent in a baby carrier or sling, car seat or capsule, stroller or pram, highchair, bouncer, jolly jumper or play pen
5. **Watching** television programs, videos/internet clips or movies on a television, computer or portable/mobile device such as tablet or smartphone
6. **Playing games**, looking at photos, or video chatting (e.g. FaceTime, Zoom, Skype) on screen-based devices such as a computer or laptop, videogame console, iPad, tablet, or smartphone

If your baby *did not* do any one of these activities over the 24-hour period, you do not have to mark any times for that activity.

If you need assistance, please contact the Project Coordinator (Denise Brookes) on 0409 053 374. Thank you!

Please **circle** which day this log has been completed on: MONDAY TUESDAY WEDNESDAY THURSDAY FRIDAY SATURDAY SUNDAY

Please record the **date** this log has been completed on: ____ / ____ / 2021 Is this a childcare day? ☐ Yes ☐ No

	MORNING																															
**ACTIVITY**	**5 AM**				**6 AM**				**7 AM**				**8 AM**				**9 AM**				**10 AM**				**11 AM**				**12 PM**			
**What is your baby doing?**	00	15	30	45	00	15	30	45	00	15	30	45	00	15	30	45	00	15	30	45	00	15	30	45	00	15	30	45	00	15	30	45
1. Sleeping																																
2. Tummy time																																
3. Supervised active play																																
4. Restrained time																																
5. Watching TV, videos/internet clips or movies																																
6. Playing games, looking at photos, or video chatting on screen-based devices																																

	AFTERNOON / EVENING																															
**ACTIVITY**	**1 PM**				**2 PM**				**3 PM**				**4 PM**				**5 PM**				**6 PM**				**7 PM**				**8 PM**			
**What is your baby doing?**	00	15	30	45	00	15	30	45	00	15	30	45	00	15	30	45	00	15	30	45	00	15	30	45	00	15	30	45	00	15	30	45
1. Sleeping																																
2. Tummy time																																
3. Supervised active play																																
4. Restrained time																																
5. Watching TV, videos/internet clips or movies																																
6. Playing games, looking at photos, or video chatting on screen-based devices																																

	NIGHT																															
**ACTIVITY**	**9 PM**				**10 PM**				**11 PM**				**12 AM**				**1 AM**				**2 AM**				**3 AM**				**4 AM**			
**What is your baby doing?**	00	15	30	45	00	15	30	45	00	15	30	45	00	15	30	45	00	15	30	45	00	15	30	45	00	15	30	45	00	15	30	45
1. Sleeping																																
2. Tummy time																																
3. Supervised active play																																
4. Restrained time																																
5. Watching TV, videos/internet clips or movies																																
6. Playing games, looking at photos, or video chatting on screen-based devices																																

24-hour Sleep and Screen Time Log for Children

DIRECTIONS: Use this form to record your child's **sleep and screen time** over a 24-hour period. This log may be easier to fill out as you go.

An example is shown below:

Please **circle** which day this log has been completed on: MONDAY TUESDAY (WEDNESDAY circled) THURSDAY FRIDAY SATURDAY SUNDAY

Please record the **date** this log has been completed on: _22_ / _04_ / 2021 Is this a childcare/school day? ☐ Yes ☑ No

	MORNING																															
**ACTIVITY**	**5 AM**				**6 AM**				**7 AM**				**8 AM**				**9 AM**				**10 AM**				**11 AM**				**12 PM**			
**What is your child doing?**	00	15	30	45	00	15	30	45	00	15	30	45	00	15	30	45	00	15	30	45	00	15	30	45	00	15	30	45	00	15	30	45
1. SLEEPING	→	→	→	→	→	→	→	→	→	→																						
2. SCREEN TIME																																
a. Watching TV, videos/internet clips or movies											→	→	→																			
b. Playing games, looking at photos, or video chatting on screen-based devices																					→	→	→									
***AND** tick if they were STANDING during screen time*																					✓	✓	✓									

DETAILED INSTRUCTIONS: In the appropriate column, draw an arrow through each 15-minute time period your child's **main activity** was:

1. **Sleeping**
2. **Screen Time**
   a. **Watching** television programs, videos/internet clips or movies on a television, computer or portable/mobile device such as tablet or smartphone
   b. **Playing games**, looking at photos, or video chatting (e.g. FaceTime, Zoom, Skype) on screen-based devices such as a computer or laptop, videogame console, iPad, tablet, or smartphone

AND *for the screen time activities (2a. Watching TV…, 2b. Playing games…),* **also** tick if your child was standing.

If your child did not do any one of these activities over the 24-hour period, you do not have to mark any times for that activity.
If you need assistance, please contact the Project Coordinator (Denise Brookes) on 0409 053 374. Thank you!

Please **circle** which day this log has been completed on: MONDAY TUESDAY WEDNESDAY THURSDAY FRIDAY SATURDAY SUNDAY

Please record the **date** this log has been completed on: ____ /____ / 2021 Is this a childcare/school day? ☐ Yes ☐ No

	MORNING																														
**ACTIVITY**	**5 AM**				**6 AM**				**7 AM**				**8 AM**				**9 AM**				**10 AM**				**11 AM**				**12 PM**			
**What is your child doing?**	00	15	30	45	00	15	30	45	00	15	30	45	00	15	30	45	00	15	30	45	00	15	30	45	00	15	30	45	00	15	30	45
1. SLEEPING																																
2. SCREEN TIME																																
a. Watching TV, videos/internet clips or movies																																
b. Playing games, looking at photos, or video chatting on screen-based devices																																
***AND** tick if they were STANDING during screen time*																																

	AFTERNOON / EVENING																														
**ACTIVITY**	**1 PM**				**2 PM**				**3 PM**				**4 PM**				**5 PM**				**6 PM**				**7 PM**				**8 PM**			
**What is your child doing?**	00	15	30	45	00	15	30	45	00	15	30	45	00	15	30	45	00	15	30	45	00	15	30	45	00	15	30	45	00	15	30	45
1. SLEEPING																																
2. SCREEN TIME																																
a. Watching TV, videos/internet clips or movies																																
b. Playing games, looking at photos, or video chatting on screen-based devices																																
***AND** tick if they were STANDING during screen time*																																

	NIGHT																														
**ACTIVITY**	**9 PM**				**10 PM**				**11 PM**				**12 AM**				**1 AM**				**2 AM**				**3 AM**				**4 AM**			
**What is your child doing?**	00	15	30	45	00	15	30	45	00	15	30	45	00	15	30	45	00	15	30	45	00	15	30	45	00	15	30	45	00	15	30	45
1. SLEEPING																																
2. SCREEN TIME																																
a. Watching TV, videos/internet clips or movies																																
b. Playing games, looking at photos, or video chatting on screen-based devices																																
***AND** tick if they were STANDING during screen time*																																
